# Pelvic Belt Effects on Health Outcomes and Functional Parameters of Patients with Sacroiliac Joint Pain

**DOI:** 10.1371/journal.pone.0136375

**Published:** 2015-08-25

**Authors:** Niels Hammer, Robert Möbius, Stefan Schleifenbaum, Karl-Heinz Hammer, Stefan Klima, Justin S. Lange, Odette Soisson, Dirk Winkler, Thomas L. Milani

**Affiliations:** 1 Institute of Anatomy, University of Leipzig, Faculty of Medicine, Leipzig, Germany; 2 Department of Orthopedics, Trauma and Reconstructive Surgery, University Clinics of Leipzig, Leipzig, Germany; 3 Clinics for Orthopedics, Osteology and Pain Treatment, Kirchberg, Germany; 4 Institute of Applied Kinesiology, Department Human Locomotion, Chemnitz University of Technology, Chemnitz, Germany; 5 Department of Neurosurgery, University Clinics of Leipzig, Faculty of Medicine, Leipzig, Germany; The James Cook University Hospital, UNITED KINGDOM

## Abstract

**Introduction:**

The sacroiliac joint (SIJ) is a common source of low back pain. However, clinical and functional signs and symptoms correlating with SIJ pain are widely unknown. Pelvic belts are routinely applied to treat SIJ pain but without sound evidence of their pain-relieving effects. This case-control study compares clinical and functional data of SIJ patients and healthy control subjects and evaluates belt effects on SIJ pain.

**Methods:**

17 SIJ patients and 17 healthy controls were included in this prospective study. The short-form 36 survey and the numerical rating scale were used to characterize health-related quality of life in patients in a six-week follow-up and the pain-reducing effects of pelvic belts. Electromyography data were obtained from the gluteus maximus, biceps femoris, rectus femoris and medial vastus. Alterations of muscle activity, variability and gait patterns were compared in patients and controls along with the belts’ effects in a dynamic setting when walking.

**Results:**

Significant improvements were observed in the short-form 36 survey of the SIJ patients, especially in the physical health subscores. Minor declines were also observed in the numerical rating scale on pain. Belt-related changes of muscle activity and variability were similar in patients and controls with one exception: the rectus femoris activity decreased significantly in patients with belt application when walking. Further belt effects include improved cadence and gait velocity in patients and controls.

**Conclusions:**

Pelvic belts improve health-related quality of life and are potentially attributed to decreased SIJ-related pain. Belt effects include decreased rectus femoris activity in patients and improved postural steadiness during locomotion. Pelvic belts may therefore be considered as a cost-effective and low-risk treatment of SIJ pain.

**Trial Registration:**

ClinicalTrials.gov NCT02027038

## Introduction

The sacroiliac joint (SIJ) is the source of low back pain in 15% to 30% of patients with non-radicular pain [1–7]. Though the SIJ is structurally optimized to transmit loads between the trunk and the lower extremity [8, 9], it frequently becomes involved in painful conditions of the pelvis and lower extremity due its complex anatomy [10], consisting of synovial [9, 11–13] and fibrous regions [14, 15]. However, the SIJ is hard to identify as the specific source of low back pain [2–4, 6, 16] and is therefore underappreciated in routine examinations [3, 7, 17–19]. Moreover, several established tests to identify the SIJ as a pain generator [2–4, 6, 16, 20] are lacking in specificity or sensitivity [21]. In clinical practice, the diagnosis of SIJ pain is often made by exclusion [22] and eventually stands at the end of protracted therapeutic interventions without the anticipated success [3, 23, 24].

Furthermore, morphological or functional correlates of SIJ pain are widely unknown [25, 26]. Only few studies attempt to clarify the causal relationship between SIJ pain and pelvic or lower limb anatomy [12, 27–33] or muscle activation. Such morphological and functional data would potentially help distinguish SIJ patients from healthy controls and therefore provide a better understanding of the pathomechanisms that stand behind SIJ pain.

According to the guidelines of the international association for the study of pain, dysfunctions of the SIJ should primarily be managed non-surgically with surgical interventions being limited to treatment-refractory cases [6, 20, 34, 35]. The application of pelvic belts is one of a variety of conservative measures in the therapy of SIJ pain [36]. These belts are inexpensive and without any reported adverse side effects. However, there is limited evidence concerning the clinical efficiency of pelvic belts and the underlying mechanisms are subject to ongoing controversies [33, 36, 37].

Our present study investigates clinical and functional data of SIJ patients to healthy age-matched control subjects and immediate pain-relieving effects of pelvic belts in a dynamic setting and compares the muscle activity and gait pattern data when walking. Additionally, short-form 36 survey data were obtained in a six-week follow up to quantify belt-related health effects in SIJ patients. It was hypothesized that pelvic belts are capable of decreasing SIJ-related pain and improving health-related quality of life in SIJ patients in a six-week follow up. It was further hypothesized that pelvic belts alter muscle activity, the variability of muscle activity and gait patterns and that these changes were different in SIJ patients compared to the healthy controls.

## Material and Methods

**Fig 1:**
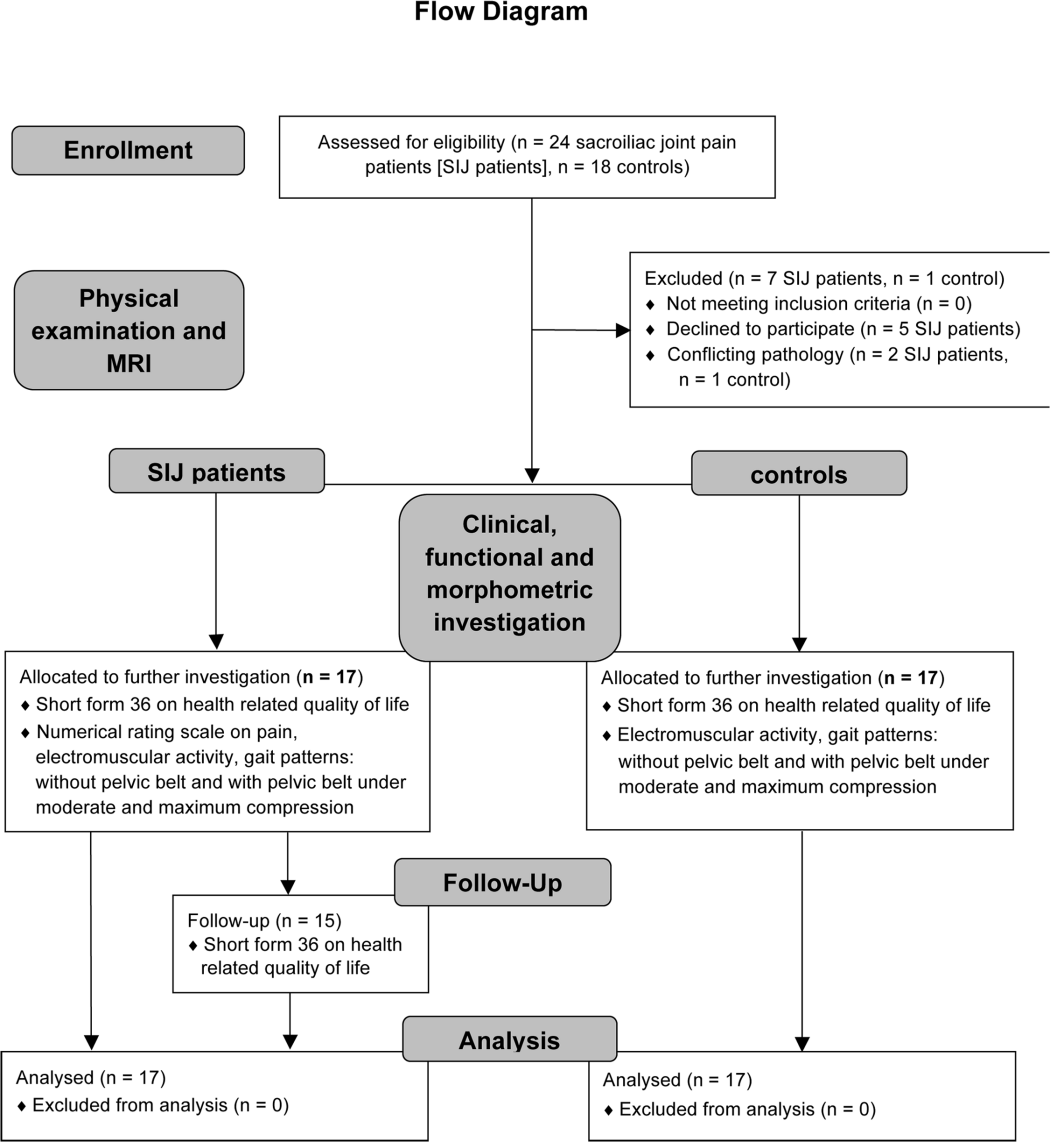
Modified CONSORT flow diagram.

**Fig 2:**
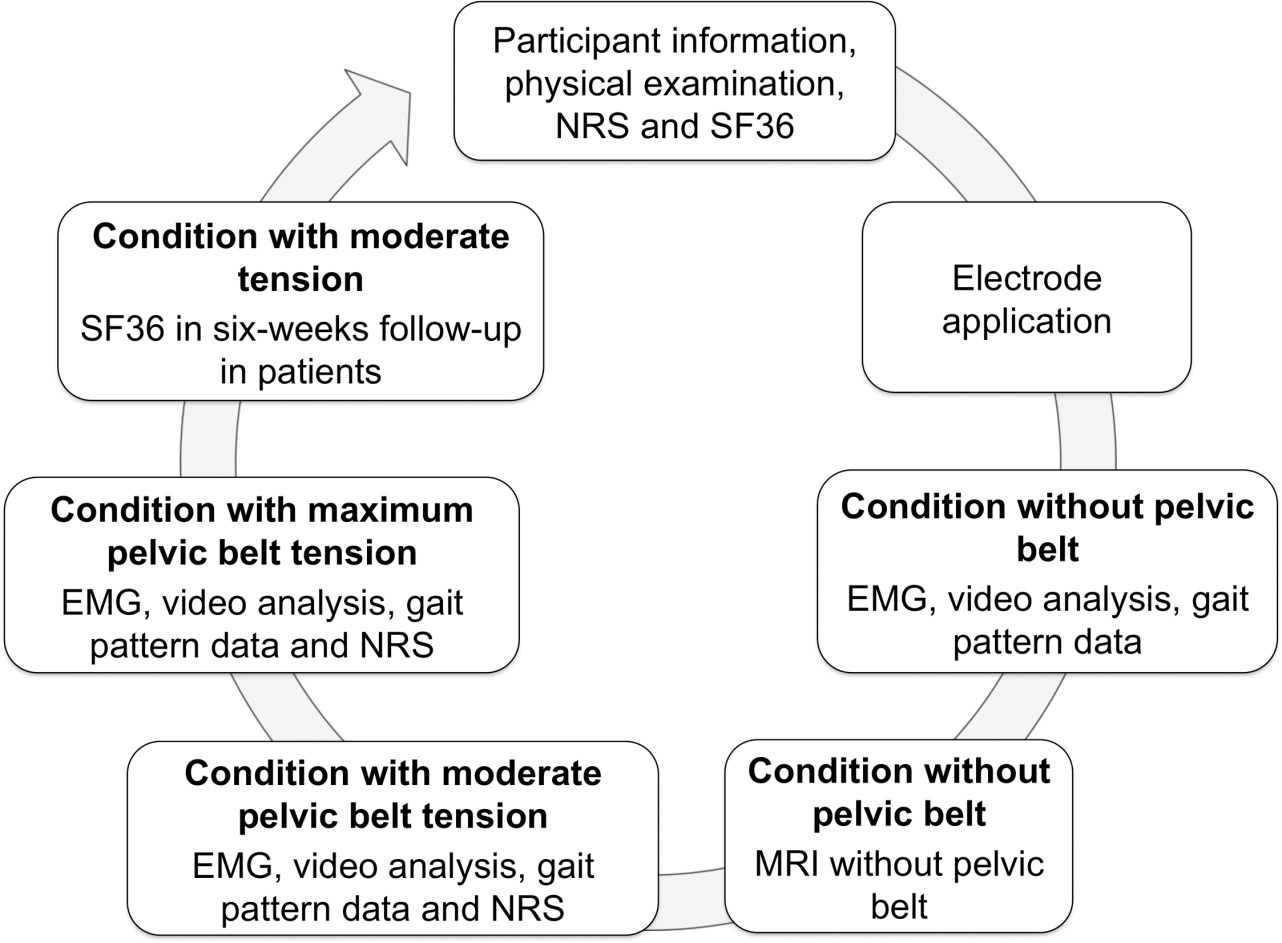
Summary of the experimental setup; COP = center of pressure recordings, EMG = electromyography data, MRI = magnetic resonance imaging.

This study was approved by the ethics committee of the University of Leipzig (number 063-11-07032011; S1 File) and registered at ClinicalTrials.gov (NCT02027038 to N.H.; S2 File). Written consent was ratified from all participants. The ethics committee approved the clinical trial protocol shown in Figs 1 and 2 before the trial began. Written consent was ratified from all participants. The principal investigator (N.H.) delayed the registration of the study until data acquisition was completed for confidentiality reasons concerning the study methods, especially the magnetic resonance with the related morphometric measurements [38]. The authors confirm that all ongoing and related trials for this intervention are registered.

### General information

The study population encompassed 24 patients suffering from pain originating from the sacroiliac joint (SIJ) and 18 age-matched controls without any history of musculoskeletal disorders, enrolled between August 2011 and December 2012 (Table 1; S1 Table). The patients were sent from several orthopedic outpatients’ clinics and were included on basis of the following criteria: pain duration of at least 12 weeks, at least three positive SIJ pain provocation tests including the thigh thrust test, compression test, the active straight leg raise test, Patrick’s sign, the Gaenslen and the Faber test [2, 6, 20] and temporal pain relief of at least 75% after intra-articular injection of anesthetics [39–41]. Additionally, patient data were recorded including patient history, medication, previous injections and physical therapy and other attempts to treat the SIJ. The exclusion criteria of the entire study population were as follows: fractures, muscular disorders, degenerative and inflammatory joint diseases, metallic implants, claustrophobia, somatoform disorders and pregnancy. All participants were interviewed regarding their health condition and medical history before they underwent a second physical examination for diagnostic confirmation. A flow diagram [42] and the study protocol are given in Figs 1 and 2, respectively, according to the STROBE guidelines [43].

**Table 1:** Summary of baseline data of patients with sacroiliac joint (SIJ) pain, pain duration and numerical rating scale (NRS) data. Mean values ± standard deviations are given. BMI = body mass index

			SIJ patients	controls
Age		[years]	45.1±11.0	43.7±19.9
Height		[m]	1.7±0.1	1.7±0.1
Weight		[kg]	73.2±11.3	68.1±9.3
BMI		[kg/m^2^]	24.9±3.4	24.2±3.9
Pain duration		[months]	54.5±49.7	
Numerical rating scale	last 2 weeks		5.0±1.9	
	no belt		4.0±1.8	
	moderate tension		3.4±2.1	
	maximum tension		4.0±1.9	

Three Tesla MRI (MAGNETOM TRIO, Siemens AG, Erlangen, Germany) were obtained from the lumbar spine, the entire pelvis and the SIJ as well as both hip joints from all participants to rule out inflammatory causes of SIJ pain or extra-articular pathologies that potentially cause comparable symptoms [12, 44] prior to further investigations. Further information on the MRI setup is published elsewhere [38]. Pelvic belt effects (SacroLoc, Bauerfeind AG, Zeulenroda-Triebes, Germany) were investigated under the following conditions: without pelvic belt application, under moderate tension and maximum tension of the pelvic belt. The magnitude of moderate tension was adapted by the participants as being suitable for everyday situations, according to the manufacturer. Maximum tension was defined as tolerable tautness without perceiving pelvic belt-related pain or discomfort in the standing position. Each pelvic belt was exclusively used for one participant. Four different clothing sizes of the belt were available, being adapted depending on the pelvic circumference of each participant.

### Short Form 36 health survey (SF36) and Numerical Rating Scale (NRS)

All participants were interviewed on the day of investigation without belt application regarding their health related quality of life by means of the SF36 health survey [45, 46] (Fig 1). Patients were additionally surveyed in the follow-up with the SF36 (version 2) after six weeks of pelvic belt use under moderate tension. For this follow-up period, the patients were asked to wear the belt at least three hours daily at their own discretion. For comparison to the general (German) population, the values of all SF36 subscales were transformed into z-values according to Bullinger et al. [46]. An 11-point NRS was used to survey the patients on their SIJ pain intensity. The patients were surveyed retrospectively for the two-week period before the study and prospectively on the investigation day without applying the pelvic belt and with the pelvic belt under moderate and under maximum tension.

### Surface electromyography (EMG), video capturing and plantar force distribution

**Fig 3:**
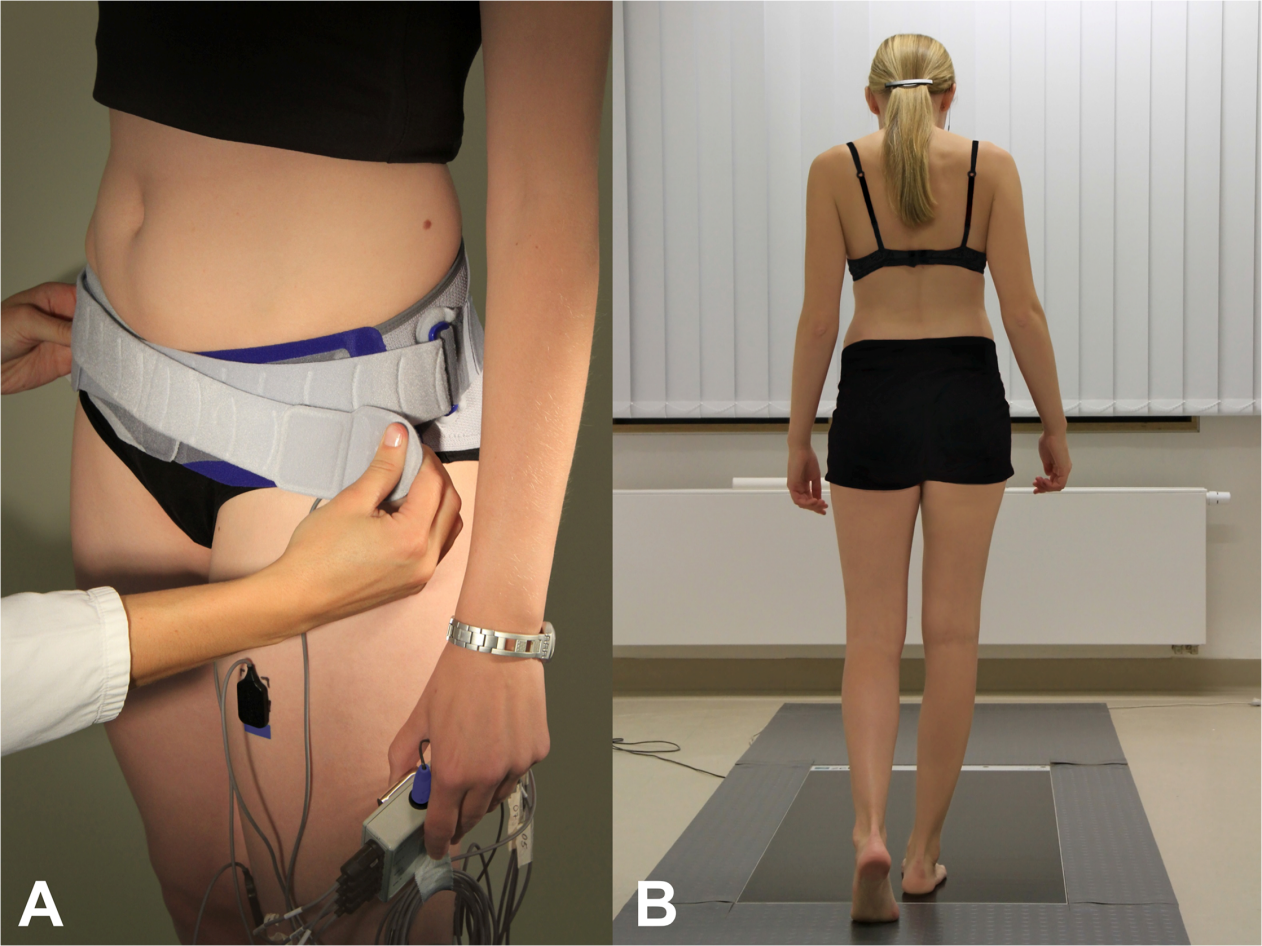
A: Application of a pelvic belt (SacroLoc, Bauerfeind AG, Zeulenroda-Triebes, Germany) to a 26 year-old female control. B: Recording of gait pattern data on force plates in all participants without pelvic belt, under moderate and maximum tolerable compression.

Surface EMG were obtained from the muscles of the dominant leg of all participants simultaneously with video capturing and gait pattern data when walking. The biceps femoris, the gluteus maximus, the medial vastus, the rectus femoris and the tibialis anterior muscle were included with the reference electrode placed at the lateral malleolus of the respective side. The conditions without a pelvic belt and with a pelvic belt under moderate and maximum tension were investigated, recording ten cycles each (Figs 1 and 3A). The EMG data were captured at 1000 Hz according to SENIAM recommendations [47] with a Bagnoli-8 EMG system and a band pass filter ranging from 5 to 450 Hz (Delsys Inc., Boston, MA, USA). In the EMG datasets, the single walking step of interest of each walking cycle was identified manually by means of the video data (Handycam DCR-PC120, Sony Corp., Tokyo, Japan) and the activity of the tibialis anterior muscle. Then, the root mean square (RMS) was calculated from the signal amplitude of each muscle in the respective step of each cycle, using RStudio software (Version 0.97.551, RStudio Inc., Boston, MA, USA). The resulting RMS of the ten walking cycles (c) of each participant were averaged to compute the mean values ($\bar{\text{RMS}}$) as follows:

$${\bar{\text{RMS}}}_{\text{no belt}}\;=\;\frac{1}{\text{c}}\sum_{\text{i}=1}^{\text{c}}{\text{RMS}}_{\text{no belt}}\;;\text{c}=10,$$

$${\bar{\text{RMS}}}_{\text{moderate tension}}\;=\;\frac{1}{\text{c}}\sum_{\text{i}=1}^{\text{c}}{\text{RMS}}_{\text{moderate tension}}\;;\text{c}=10,$$

and

$${\bar{\text{RMS}}}_{\text{maximum tension}}\;=\;\frac{1}{\text{c}}\sum_{\text{i}=1}^{\text{c}}{\text{RMS}}_{\text{maximum tension}}\;;\text{c}=10.$$

The mean RMS of the condition without pelvic belt (${\bar{\text{RMS}}}_{\text{no belt}}$) was set as the reference value. Relative alterations of RMS activity related to pelvic belt application were then calculated for each participant in the conditions with pelvic belt under moderate (ΔRMS_moderate tension_) and maximum tension (ΔRMS_maximum tension_):

$${\Delta \text{RMS}}_{\text{moderate tension}}\;=\;\frac{{\bar{\text{RMS}}}_{\text{moderate tension}}-{\bar{\text{RMS}}}_{\text{no belt}}}{{\bar{\text{RMS}}}_{\text{no belt}}}\;\times \;100\%,$$

and

$${\Delta \text{RMS}}_{\text{maximum tension}}\;=\;\frac{{\bar{\text{RMS}}}_{\text{maximum tension}}-{\bar{\text{RMS}}}_{\text{no belt}}}{{\bar{\text{RMS}}}_{\text{no belt}}}\;\times \;100\%.$$

If single ΔRMS values of the respective condition under moderate and maximum tension varied more than 200% from the ${\bar{\text{RMS}}}_{\text{no belt}}$ in a participant, the data of this muscle was assumed to be erroneous and this data was excluded from further evaluation. The mean values and standard deviations of the ΔRMS were then computed for the SIJ patients and the controls. Furthermore, the variability of the RMS data was computed for each of the muscles of each participant under each condition of pelvic belt application before the mean values and standard deviations of muscle variability were summarized for SIJ patients and controls.

Gait parameters such as walking speed, cadence and step width were measured using a capacitive pressure distribution platform measuring 153.0 cm x 60.5 cm (FDM 1.5, zebris Medical GmbH, Isny, Germany; 11,264 sensors, 1.22 sensors/cm^2^; Fig 3B). The platform’s temperature range was 15°C to 40°C and the pressure range was 1 N/cm^2^ to 120 N/cm^2^ with an accuracy of ±5%. The data were collected at a frame rate of 100 Hz. The pressure distribution platform was embedded in a wooden platform measuring one by three meters to minimize any targeting of the subjects to the measuring plate. The participants were asked to walk over the platform barefoot and at a self-selected pace. Furthermore the participants were encouraged to fix their view on a target at eye level behind the platform to prevent an untypical gait pattern attributable to step size adaption or speed up and braking operations on the platform. Due to the dimensions of the platform, two or more consecutive steps of each participant could be measured within each trial.

### Statistical analysis

The data were analyzed statistically be means of Microsoft Excel 2010 (Redmond, WA, USA) and SPSS version 20.0 (Armonk, NY, USA). Normal distribution was determined with the Kolmogorow-Smirnow-Test or with the Shapiro–Wilk test. Baseline characteristics and the SF36 subscales were evaluated with Student’s t-test or the Mann-Whitney U-test. A non-parametric test (Friedman test) was used to investigate alterations in the NRS values. The RMS values, their variations and the gait pattern data were compared between SIJ patients and controls with the Mann-Whitney U-test. The Wilcoxon signed-rank test and the Friedman-test with post-hoc analyses were used to compare the data of the different conditions of belt application within the SIJ patients and the controls for $\bar{\Delta }\text{RMS}$ and RMS variability, respectively. *P*-values of 5% or less than were considered as statistically significant. Effect size and power analyses on the NRS, the SF36 physical and the SF36 mental summary scores were calculated post hoc [48] given the limited number of SIJ patients available for this study. G*Power software (V 3.1.9.2, Kiel, Germany) was used for this purpose. Moreover, due to the small sample size, the resulting *p*-values for multiple comparisons remained unadjusted to minimize the risk of β-type errors [49].

## Results

Data of 17 patients suffering from SIJ pain (10 ♀, 7 ♂) and 17 healthy controls (11 ♀, 6 ♂) were included in this prospective investigation (Fig 1). The patient mean age was 45.1 ± 11.0 (mean value ± standard deviation) years and the mean body mass index (BMI) was 24.9 ± 3.4 kg/m^2^. Controls had a mean age of 43.7 ± 19.9 years and a mean BMI of 24.2 ± 3.9 kg/m^2^. Age, body height, weight and BMI did not vary significantly between SIJ patients and controls. The duration of SIJ pain in the patient group averaged 54.5 ± 49.7 months. The NRS scores and further baseline data are given in Table 1 and S1 Table. Seven SIJ patients and one control were excluded for the following reasons: claustrophobia in MRI (five SIJ patients) and conflicting pathology after physical examination (1 SIJ patient and 1 control).

Another SIJ patient was excluded after interpretation of the MRI records showed a conflicting gynecological pathology. The dominant side proved to be the (more severely) affected side in 10 of the 17 SIJ patients (58.8%) with the corresponding muscle data being evaluated in this study. The RMS data, RMS variability data and the gait analysis data were not normally distributed.

### Pelvic belt application is linked to significantly improved SF36 physical subscales

**Fig 4:**
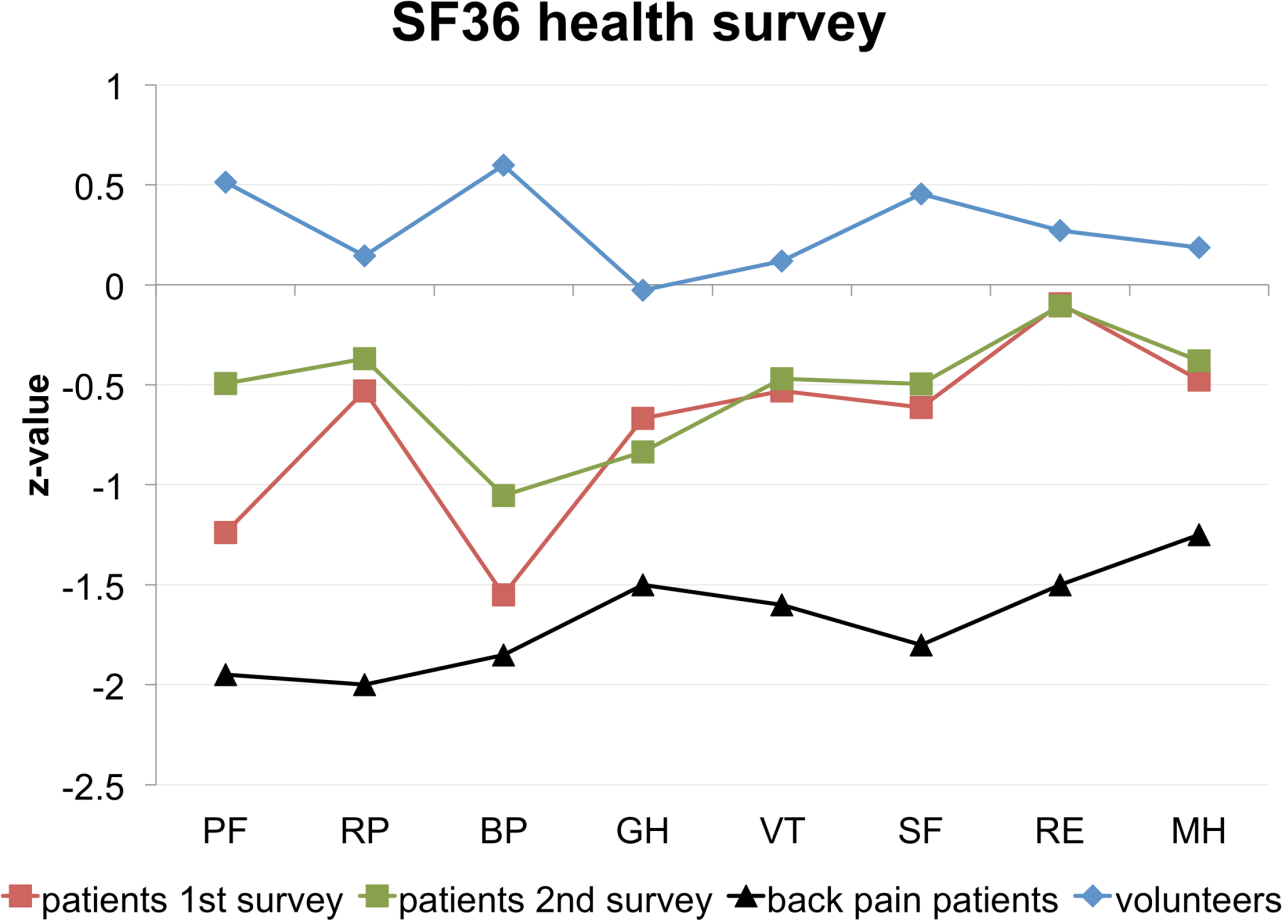
Short-form 36 survey of patients with sacroiliac joint pain, controls and comparison to low back pain patients: Six-week belt application improved health-related quality of life in the patients. PF = physical role functioning, RP = role physical, BP = bodily pain, GH = general health perceptions, VT = vitality, SF = social role functioning, RE = role emotional functioning, MH = mental health.

On the investigation day, the response rate of the SF36 survey was 17/17 (100%) for SIJ patients and 17/17 (100%) for the controls. The response rate of the six-week follow-up SF36 for SIJ patients was 15/17 (88%). The control group was similar to the German average population, as indicated by the z-transformed SF36 values close to *z* = 0 (Fig 4, S2 Table). In the survey taken prior to pelvic belt application, the SIJ patients were more constrained in the physical scales than in the mental and social ones (Table 2). This finding is supported by the negative z-values in the physical functioning and bodily pain subscores shown in Fig 4, being more than one standard deviation lower (*Δz* < -1) than the healthy reference (*z* = 0). Also, the SIJ patients were less constrained in the mental and social subscales (*Δz* ~ -0.5; Fig 4), being close to the average SF36 population. Prior to belt application, SIJ patients scored significantly lower than the age-matched healthy controls in the physical functioning (*p* < 0.001; S3 Table), role functioning physical (*p* = 0.04), bodily pain (*p* < 0.001), physical summary (*p* < 0.001), and also in vitality (*p* = 0.011) and social functioning (*p* = 0.004), as indicated by the SF36. Comparison the SF36 physical summary of SIJ patients without belt application and controls revealed an effect size ranging between 1.89 and 3.35 and a statistical power of 1.00 (Table 3). The effect size of the SF36 mental summary between SIJ patients and controls was 0.14 with a post-hoc power of 0.10.

**Table 2:** Short Form 36 (SF36) transformed scores of patients with sacroiliac joint (SIJ) pain prior to pelvic belt application (pre) and in a six-weeks follow up with pelvic belt application (post). Mean values ± standard deviations are given.

	Effect size (d)	Power
**Numerical rating scale**		
Last 2 weeks vs. no belt	0.43	0.52
Last 2 weeks vs. moderate tension	0.73	0.89
Last 2 weeks vs. maximum tension	0.46	0.57
No belt vs. moderate tension	0.35	0.40
Moderate vs. maximum tension	0.31	0.34
**Short-Form 36 health survey**		
Physical summary pre vs. post	0.66	0.83
Physical summary controls vs. patients pre	3.35	1.00
Physical summary controls vs. patients post	1.89	1.00
Mental summary pre vs. post	0.24	0.24
Mental summary controls vs. patients pre	0.14	0.10
Mental summary controls vs. patients post	0.46	0.35

In the six-week follow-up of the SIJ patients, an improvement was observed in most of the subscores (Fig 4). Physical functioning and bodily pain improved the most with z-value increases of +0.75 and +0.50, respectively (Fig 4; S2 Table). The role functioning physical, general health and the mental subscores remained largely unchanged in the follow up (-0.14 < *Δz* < +0.14; Fig 4; S2 Table). Statistical comparison to the SF36 data obtained from the participants prior to belt application showed that significant differences remained in the role functioning physical (*p* < 0.001), bodily pain (*p* < 0.001) and physical summary scores (*p* < 0.001; S4 Table). However increases were observed in these scores of SIJ patients in the six-week follow up, in physical functioning (*p* = 0.002), bodily pain (*p* = 0.006) and in the physical summary score (*p* = 0.013; Fig 4; Table 2). An effect size of 0.66 and a statistical power of 0.83 were determined in the post-hoc analyses for the SF36 physical summary improvements in the SIJ patients (Table 3).

**Table 3:** Post-hoc effect size and power analyses on the Numerical Rating Scale and Short-Form 36 physical summary and mental summary.

	Effect size (d)	Power
Numerical rating scale		
Last 2 weeks vs. no belt	0.43	0.52
Last 2 weeks vs. moderate tension	0.73	0.89
Last 2 weeks vs. maximum tension	0.46	0.57
No belt vs. moderate tension	0.35	0.4
Moderate vs. maximum tension	0.31	0.34
		
Short-Form 36 health survey		
Physical summary pre vs. post	0.66	0.83
Physical summary controls vs. patients pre	3.35	1
Physical summary controls vs. patients post	1.89	1
Mental summary pre vs. post	0.24	0.24
Mental summary controls vs. patients pre	0.14	0.1
Mental summary controls vs. patients post	0.46	0.35

A comparison to the chronic low back pain reference group showed that health perceptions of SIJ patients are better on the examination day as well as in the six-week follow-up: SIJ patients scored at least one standard deviation (*Δz* > 1) higher than the chronic low back pain reference group in physical role functioning, vitality, social role functioning and role emotional functioning subscores, and were closer to the healthy controls than to the low back pain group (Table 4).

**Table 4:** Comparison of the alterations of muscle activation between SIJ patients and controls related to moderate and maximum tension.

	ΔRMS_moderate tension_			ΔRMS_maximum tension_		
	SIJ patients	controls	*p*	SIJ patients	controls	*p*
			* *			* *
**Muscle**						
Biceps femoris	8.9±19.0	3.5±15.6	*0*.*505*	10.5±23.9	0.1±21.0	*0*.*170*
Gluteus maximus	14.8±26.7	13.8±38.7	*0*.*675*	27.5±45.7	10.6±49.2	*0*.*473*
Rectus femoris	-19.5±16.4	2.9±27.0	***0*.*023***	11.5±62.1	1.8±34.0	*0*.*484*
Medial vastus	-14.1±20.7	5.3±17.9	*0*.*660*	-14.4±15.2	7.2±34.6	*0*.*740*

### Pelvic belt application is potentially attributed to decreased pain intensity in SIJ patients

**Fig 5:**
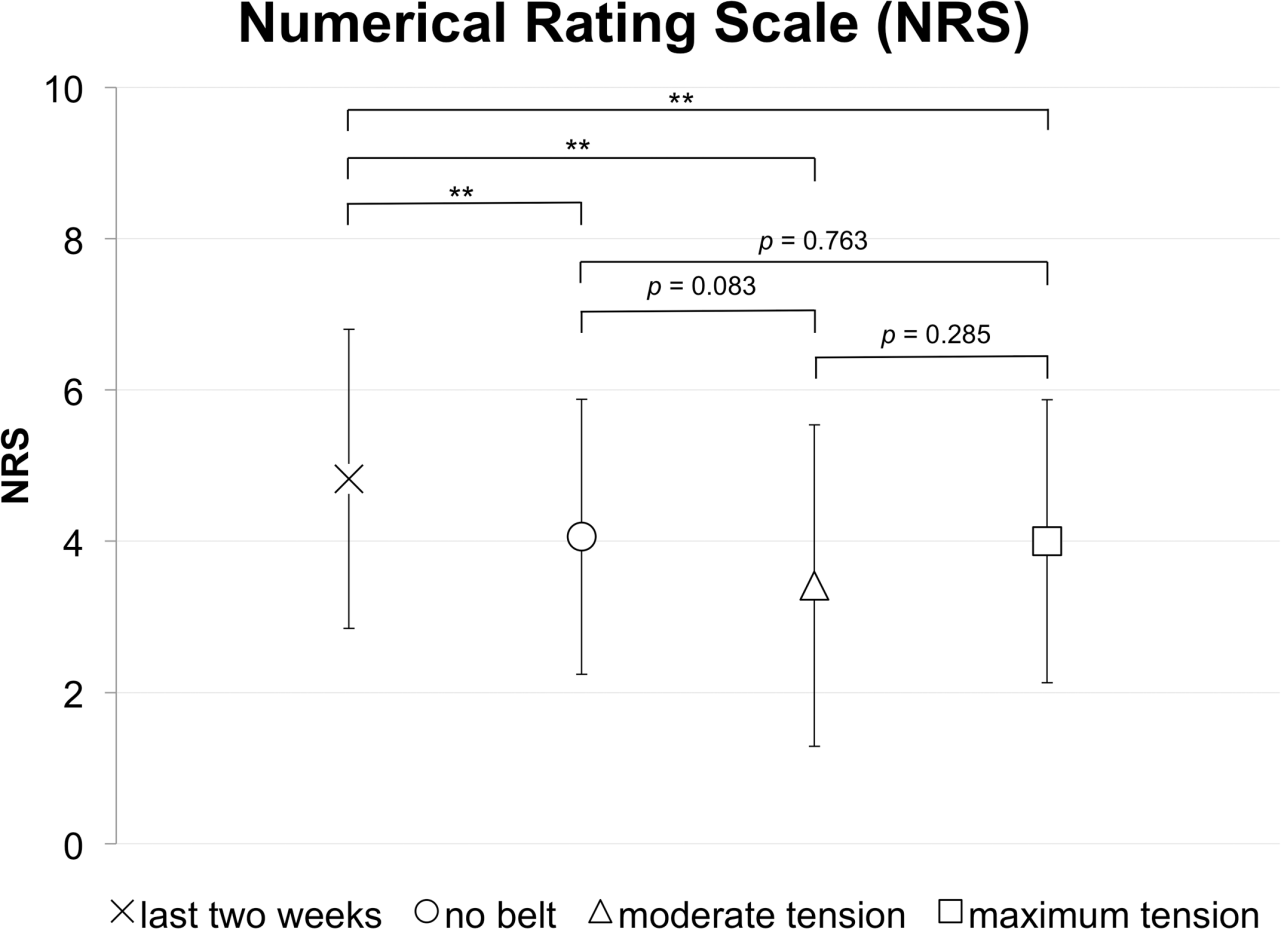
Numerical Rating Scale of patients with sacroiliac joint pain in the investigation day without and with pelvic belt application: Pelvic belt application is related to significant decreases of pain perceptions, as compared to the last two-week perception without belt application. ** *p* < 0.01.

The SIJ patients’ NRS was 5.0 ± 1.9 for the two-week period before the investigation day and decreased significantly on the investigation day: 4.0 ± 1.8 (*p* = 0.002) without using a pelvic belt but after physical examination, 3.4 ± 2.1 (*p* < 0.001) under moderate and 4.0 ± 1.9 (*p* = 0.001) under maximum belt tension, respectively (Fig 5; Table 1; S1 Table). Comparison of patients’ NRS on the investigation day revealed that the NRS were non-significantly different, comparing no belt to moderate tension (*p* = 0.083), no belt to maximum tension (*p* = 0.763) and moderate to maximum tension (*p* = 0.285), though no belt to moderate tension showed a clear tendency. Post-hoc effect size and power analyses showed small effect sizes and a lack of statistical power for these comparisons (Table 3) except for the comparison of the two-week period before the investigation and the pelvic belt applied under moderate tension (effect size: 0.73; post-hoc power: 0.89).

### Rectus femoris activity decreases significantly in SIJ patients under pelvic belt application

**Fig 6:**
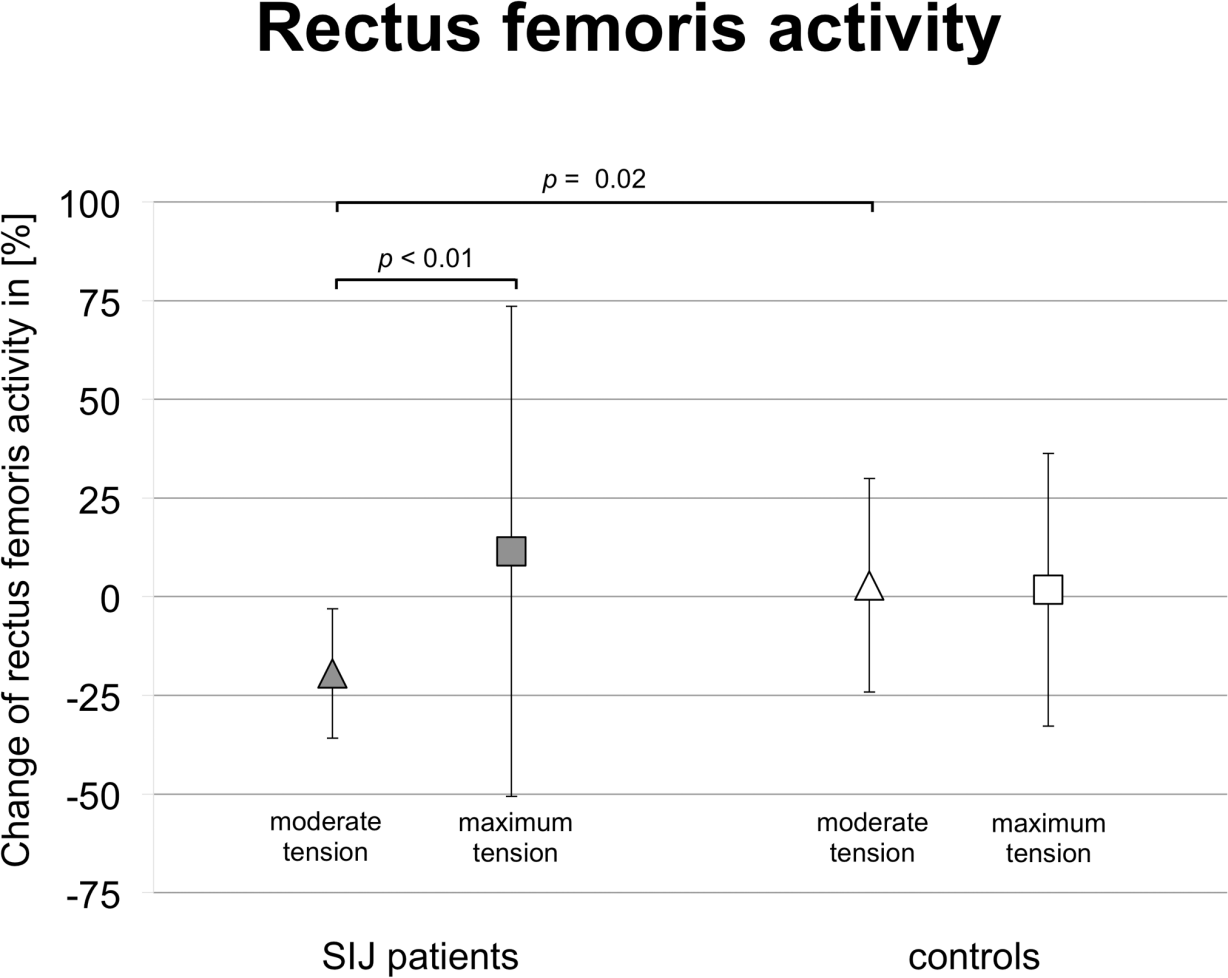
Summary of differences of muscle activation between patients with sacroiliac joint pain and controls and pelvic belt-related effects.

EMG data of ten SIJ patients and 17 controls were included for evaluating the RMS. Comparison of the $\bar{\Delta }\text{RMS}$ data revealed that pelvic belt application increased muscle activity in the controls, whereas the muscle activity of the SIJ patients increased or decreased under moderate or maximum belt tension (Fig 6; Table 4, S5 Table). With the belt moderately applied, the $\bar{\Delta }\text{RMS}$ of the rectus femoris was significantly lower in SIJ patients (Table 4), as compared to the controls (*p* = 0.02), which was not the case under maximum tension. Comparison of $\bar{\Delta }\text{RMS}$_moderate tension_ and $\bar{\Delta }\text{RMS}$_maximum tension_ in the SIJ patient group further showed that there were significantly different changes in the activation of the rectus femoris (*p* < 0.01).

### RMS variability is similar in SIJ patients and controls and largely unaltered by pelvic belt application

The comparison of the RMS variability showed that variability was always non-significantly larger in the SIJ patients than in the controls. The only exception was the RMS variability of the gluteus maximus under moderate tension, being non-significantly larger in the controls (S6 Table). Pelvic belt application caused minute changes in the RMS variability in the participants (S7 Table).

### Pelvic belt application increases cadence and gait velocity in SIJ patients and in controls

**Fig 7:**
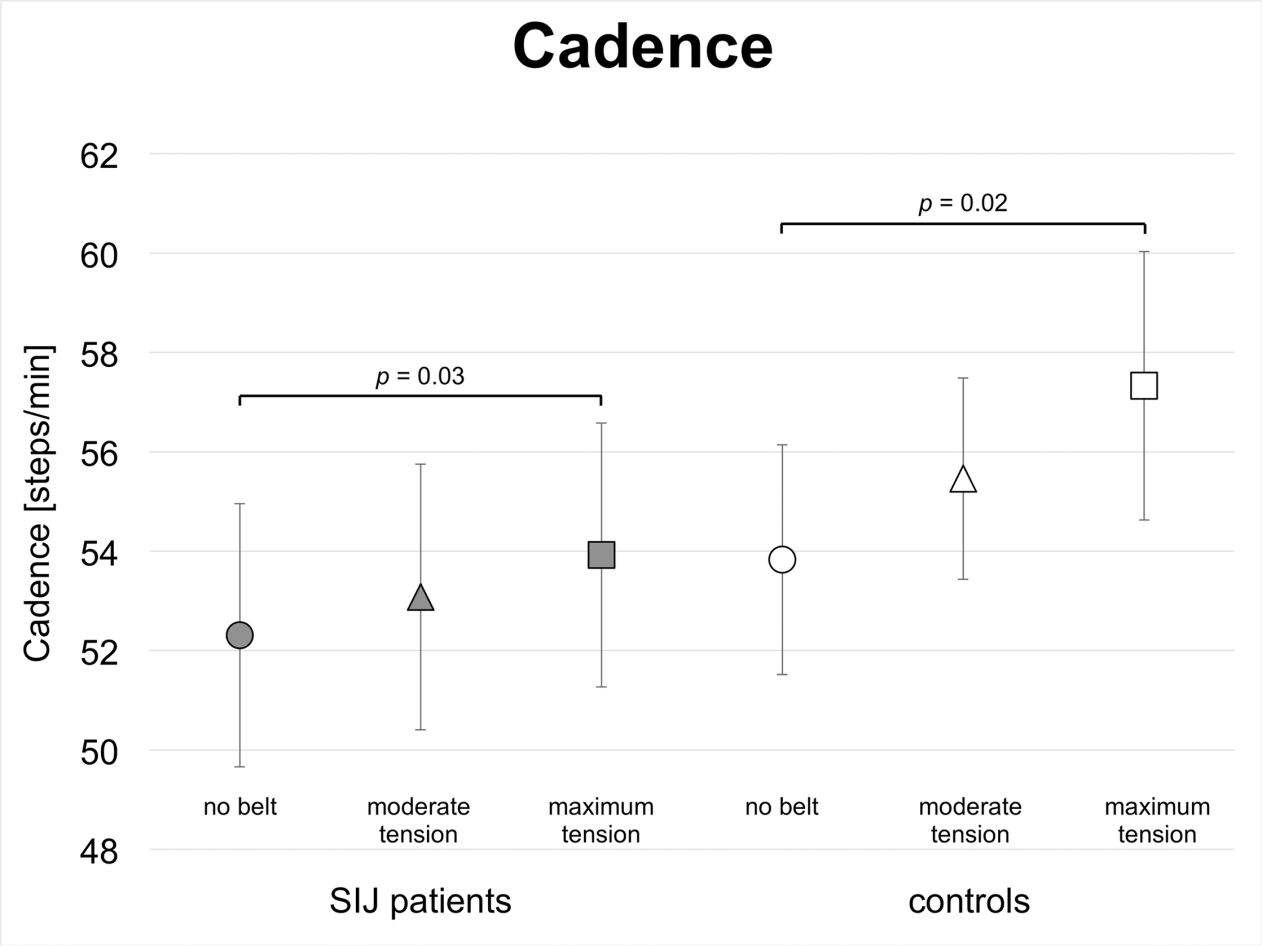
Gait analyses of patients with sacroiliac joint pain and healthy controls without and with pelvic belt application: Cadence: ○ = no pelvic belt; Δ = pelvic belt application with moderate tension; □ = pelvic belt application with maximum tension.

**Fig 8:**
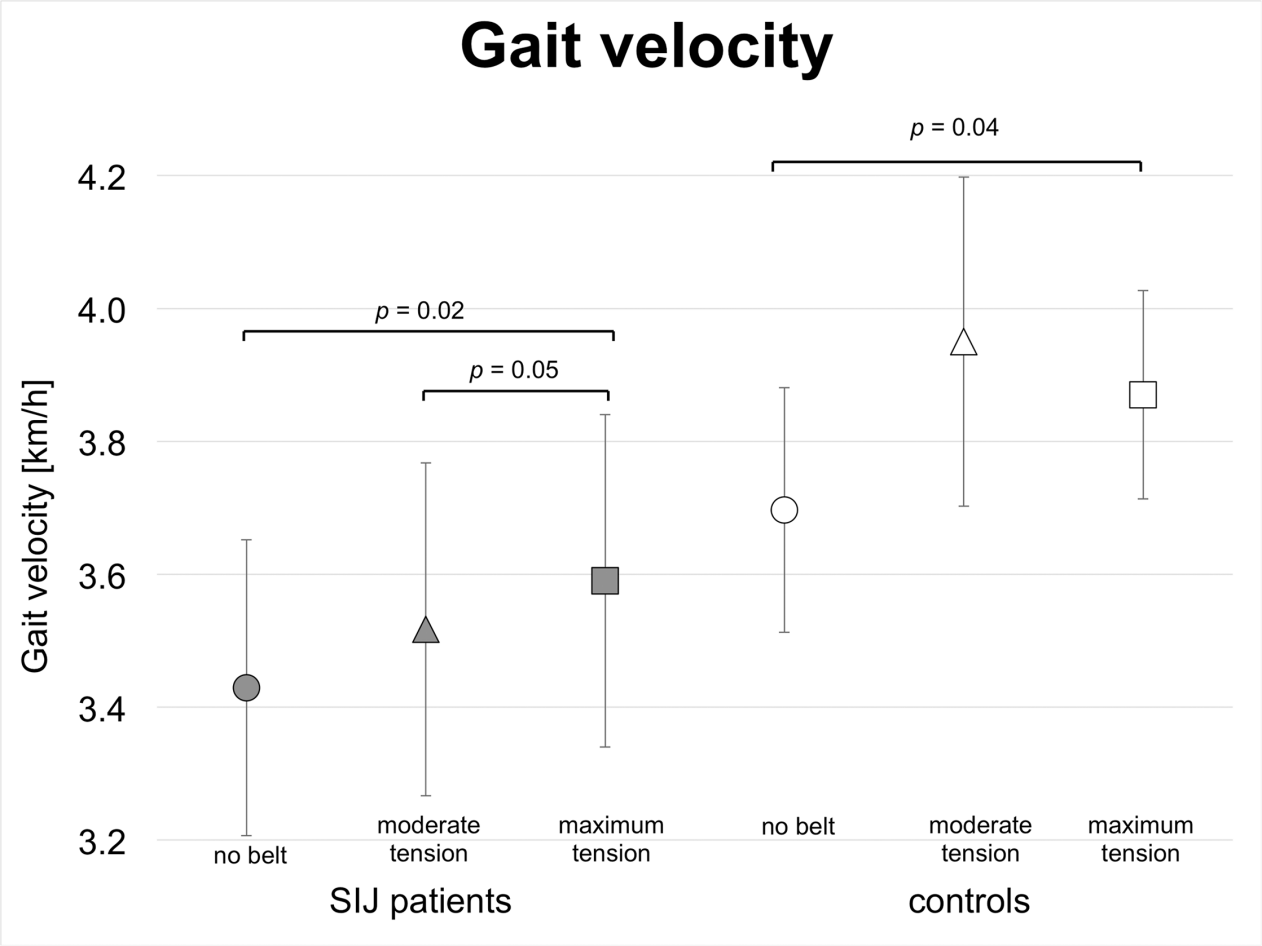
Gait analyses of patients with sacroiliac joint pain and healthy controls without and with pelvic belt application: Gait velocity; ○ = no pelvic belt; Δ = pelvic belt application with moderate tension; □ = pelvic belt application with maximum tension.

Greater belt tension increased cadence and gait velocity in SIJ patients and in controls (Figs 7 and 8; Table 5). Cadence was significantly different under moderate tension between SIJ patients (53.1 steps/min) and controls (55.5 steps/min, *p* = 0.03) and tended to be different under maximum tension (cadence_SIJ patients_ = 53.9 steps/min, cadence_controls_ = 57.3 steps/min; *p* = 0.09). Step width tended to be larger in SIJ patients (10.1 cm) than in controls (8.1 cm; *p* = 0.09) without belt application.

**Table 5:** Comparison of gait pattern data of SIJ patients and controls without pelvic belt application, under moderate and maximum belt tension.

Gait pattern data	no belt			moderate tension			maximum tension		
	SIJ patients	controls	*p*	SIJ patients	controls	*p*	SIJ patients	controls	*p*
Cadence [steps/min]	52.3±5.0	53.8±4.4	*0*.*985*	53.1±5.0	55.5±3.8	***0*.*034***	53.9±5.0	57.3±5.1	***0*.*088***
Gait velocity [km/h]	3.4±0.4	3.7±0.3	*0*.*823*	3.5±0.5	4.0±0.5	*0*.*149*	3.6±0.5	3.9±0.3	*0*.*122*
Step width [cm]	10.1±3.1	8.1±2.9	***0*.*091***	9.8±2.8	8.8±2.5	*0*.*325*	9.9±2.1	8.8±2.0	*0*.*213*

The inner-group comparison showed that within the SIJ patient group, cadence and gait velocity increased significantly, comparing the cadence under maximum tension (53.9 steps/min) to moderate tension (53.1 steps/min; *p* = 0.02) and to the cadence without the pelvic belt (52.3 steps/min; *p* = 0.05; Figs 7 and 8;,Table 6). Gait velocity increased significantly from 3.4 km/h under moderate tension to 3.6 km/h under maximum tension (*p* = 0.03). In the controls and under maximum tension, cadence (57.3 steps/min) and gait velocity (3.9 km/h) increased significantly, as compared to the cadence (53.8 steps/min; p = 0.04) and gait velocity (3.7 km/h; p = 0.02; Fig 8 and Tables 5 and 6) without belt.

**Table 6:** Inner-group comparison of the gait pattern data within SIJ patients or controls without pelvic belt application, under moderate and maximum tension (*p*-values refer to the data given in Table 5).

Gait pattern data	no belt: moderate tension		no belt: maximum tension		moderate: maximum tension	
*p-*value	SIJ patients	controls	SIJ patients	controls	SIJ patients	controls
Cadence [steps/min]	0.216	0.542	**0.020**	**0.038**	**0.048**	0.146
Gait velocity [km/h]	0.496	0.193	**0.029**	**0.021**	0.104	0.129
Step width [cm]	0.593	0.726	0.593	0.726	0.593	0.726

## Discussion

This is the first study that investigates SIJ-related pain and the effects of pelvic belts in a dynamic and case-controlled setting. The SIJ is a major target in the treatment of low back pain with a 500% increase of the costs related diagnosing and treating SIJ pain in the last decade [50–52]. The primary aim of this study was to quantify pain-relieving effects of pelvic belts in SIJ patients and to investigate belt-related alterations in muscle activity and gait parameters when walking. The second aim was to identify differences in muscle activity and gait parameters between SIJ patients and controls.

### Pelvic belts significantly improve health-related quality of life and are potentially attributed to decreased sacroiliac joint-related pain

The SF36 confirms that SIJ patients score lower in the physical subscores than the age-matched controls, and to a lesser extent also in the mental and social subscores (Fig 4). In the six-week follow up of the SIJ patients, most of their subscores were closer to the healthy reference population than to the low back pain group, especially in the physical functioning, role physical and bodily pain (Fig 4; S2 Table). The effect sizes of the comparison on the SF36 physical summary and the respective post-hoc power analysis underline the finding that pelvic belts are capable of improving health-related quality of life in patients with SIJ dysfunction. Comparable positive effects on the health-related quality of life have been reported when fusing the SIJ surgically [53]. The SIJ patients in our study were, however, less affected as to the mental and social subscores despite longer pain duration in our study (54.5 months), as compared to their study (31.2 months). These findings confirm the choice of our inclusion and exclusion criteria being valid to investigate pelvic belt effects on SIJ patients. The controls were equal to or scored slightly higher than the general SF36 population in all subscores, which may be attributed to the absence of musculoskeletal disorders and to their young mean age. In conclusion, the immediate pain-relief and an improvement in health-related quality of life, associated with the application of pelvic belts, can be demonstrated in patients with SIJ dysfunction.

Evaluation of the NRS data of the SIJ patients reveals that pain intensity is significantly lower on the day of investigation with or without belt application, as compared to the condition two weeks prior (Fig 5; Table 1). The initial NRS drop after examining the patients without belt application may likely be attributed to aspects of iatrogenic health improvement by touching the patient, to a recall bias or to the patients’ expectations on the pain-relieving effects of pelvic belts [54, 55]. These expectations may be interpreted to be increasingly met under maximum and extensively fulfilled under moderate belt tension, as indicated by constant or decreasing NRS scores, respectively [56, 57]. However, this interpretation is largely hypothetical, though it reflects the physical subscales of the SF36. The minute and statistically non-significant changes and the small effect sizes indicate that the sample size is underpowered to prove clinical relevance [58] except for the comparison between the condition two weeks prior to the study and the condition with the belt under moderate application.

Similar decreases in SIJ-related pain intensity have been reported with manipulative techniques [59, 60]. NRS pain declines of more than 50% can be reached in SIJ patients when injecting local anesthetics into the SIJ cavity [22, 61]. These effects, however, are temporal and potentially accompanied by adverse side effects [62]. Surgical fusion [63–66] and denervation procedures [67–71] potentially provide long-lasting and pain-relieving effects. However, these procedures are expensive, they have high complication rates and there is limited evidence for their long-term outcome [52, 72]. It is therefore a wide consensus that surgical interventions should be limited to patients with failed non-surgical treatment [72]. Moreover, it was shown recently that less than 50% of patients with surgical intervention to the SIJ return to work [52]. Peripheral nerve stimulation may become another minimally invasive option in the treatment of sacroiliac joint dysfunction [73–75]. However, long-term results are pending. Therefore, in order to gain more insight into the effects of pelvic belts on pain perceptions and health-related quality of life we surveyed SIJ patients with the SF36 questionnaire, yielding the improvements especially in the especially physical subscales.

### Pelvic and limb muscle activity and their variability are largely unaltered by SIJ pain or pelvic belt application with the exception of the rectus femoris when walking

Muscle activity decreases on a significantly different level are observed for the rectus femoris activity in SIJ patients when a pelvic belt was applied under moderate tension, as compared to the change induced by maximum tension (Fig 6; S5 Table). Further comparison of the condition under moderate compression reveals that rectus femoris activity decreases in SIJ patients but increases in controls and that this alteration is significantly different in SIJ dysfunction (Fig 5A; Table 4). As the rectus femoris connects the anterior inferior iliac spine with the lower extremity, the muscle is capable of exerting torsional forces onto the SIJ when the hip joint is being flexed. This anatomic relation confirms the validity of the active straight leg raise test (ASLR) as a positive pain provocation test, though it has not been investigated in this context before [28, 29, 32]. Our data further suggest that increased rectus femoris activity may be causally linked to SIJ pain and that rectus femoris activity decreases when applying a pelvic belt in the sense of form and force closure [30].

Different patterns of muscle activation are observed for the biceps femoris, gluteus maximus, rectus femoris and the medial vastus in SIJ patients and controls (Table 4). Our data confirm the findings of Hu and coworkers [29] and Jung et al. [30], who determined increased gluteus maximus activity in healthy females walking with a belt. In contrast to the data in this dynamic setting, higher biceps femoris and gluteus maximus activities have been reported in healthy controls [32] and decreasing biceps femoris activity in SIJ patients related to pelvic belt application [30]. These apparently differing results can partly be explained by the varying setup: Jung et al. [30] investigated females when standing with a different type of pelvic belt (Com-Pressor, OPTP, Canada), whereas Shadmehr and coworkers [32] obtained data from females in the lying position without a pelvic belt. However, on basis of our dynamic data with walking SIJ patients, no evidence can be found for changes in the activity of the gluteus maximus muscle discussed elsewhere [76].

Though the variability of muscle activation is always larger in SIJ patients than in controls, this difference does not reach a significant level (S6 Table). Also, pelvic belt application failed to alter the variability of muscle activation to a large extent (S7 Table). These findings indicate that painful affections of the SIJ are not necessarily accompanied by adverse changes in the neuromuscular circuits of the SIJ and that the circuits are robust against the application of external forces. Larger case numbers will help identify pelvic belt effects on muscle activation patterns in more depth. It was shown recently that SIJ manipulation is capable of altering alpha-motoneuron activity [77]. Pelvic belts potentially have similar effects on the pelvic and limb muscles, e.g. on muscle latency [32], which may be a subject of future investigations on pelvic belt effects.

### Pelvic belts improve postural steadiness of SIJ patients and controls during locomotion

With a pelvic belt applied under maximum tension, cadences and gait velocity increase significantly in SIJ patients and in controls, as compared to the condition without belt (Figs 7 and 8; Table 5). In patients, the increase of gait velocity can clearly be attributed to the additional tension exerted to the pelvis under maximum tension. These findings suggest that pelvic belts are capable of improving postural steadiness under maximum tension, but only to a minor extent under moderate tension. SIJ patients maintain their adaptability to external stimuli such as pelvic belts, which can be seen in the gait patterns.

Only sparse data can be found on gait analyses in SIJ patients in existing literature, though its use had been suggested before [78]. Two studies that used barpodometry reported altered weight distribution patterns in SIJ patients [59] and volunteers [79] after SIJ manipulation, being accompanied with altered peak pressures [79] when standing. These results could be confirmed in the experiments presented here. Recently, Mendez and coworkers proposed that force transmission to the foot is altered in patients with low back pain [80]. In the dynamic situation it can be shown that the feed-forward mechanisms are altered in both SIJ patients and controls, confirming this idea. In conclusion, pelvic belts affect gait patterns under maximum tension. The presence of chains of muscles and connective tissues may likely mechanically explain the effect of pelvic belts on the foot and their ability of increasing steadiness when walking in the sense of postural awareness.

## Summary and Conclusions

The given study has shown that pelvic belts significantly improve health-related quality of life, indicated by the SF36, and are potentially linked to decreased sacroiliac joint-related pain, indicated by declines in the NRS. Pelvic belt application is accompanied with altered rectus femoris activity when walking. Furthermore, belts improve postural steadiness. In summary, the pain-reducing effects are accompanied by an altered locomotion in healthy participants and patients with chronic sacroiliac joint dysfunction. Further research is necessary to clarify the cause-and-effect relationships between pain intensity, health-related quality of life and muscle activation patterns. Furthermore, it will be of interest in future studies whether there are responders and non-responders concerning pain decrease and muscle activation patterns.

### Limitations

Firstly, only a limited number of SIJ patients could be included due to the selection criteria. This approach, however, allowed the determination of SIJ-pain related effects. The number of patients was further reduced to keep the study setup comparable regarding the dominant foot and muscle activation data. Secondly, though the female gender, low BMI and a mean age older than 60 years have been described to be associated in the context of SIJ pain [16, 81], our study population consists of a considerable amount of males, and has a higher BMI and a lower mean age. These circumstances influence the NRS and the SF36 data. Also, the retrospective survey on the NRS scores two weeks prior to the investigation day are likely attributable to a recall bias. Thirdly, only a small number of muscles were investigated and the subsequent evaluation of the RMS data was limited to relative changes in muscle activity and the observed differences in muscle activity were minute. Especially the erector spinae and the abdominal wall muscles are known to be involved in low back pain [29, 31–33]. Taking these muscles into account is an issue to be addressed in future studies, which could potentially include gender- or side-dependent effects and the involvement of pelvic floor muscles as well.

## Supporting Information

S1 File Ethics approval including translation. (PDF)

S2 File TREND checklist. (PDF)

S1 Table Extended baseline data of patients with sacroiliac joint (SIJ) pain, pain duration and numerical rating scale (NRS) data; BMI = body mass index, l = left, r = right, RMS = root mean square, mean values ± standard deviations are given. (DOCX)

S2 Table Z-transformed values of the short form 36 (SF-36) survey obtained from healthy controls and patients with sacroiliac joint (SIJ) pain prior to pelvic belt application (pre) and in a six-week follow up (post), referenced by the German population. A *Δz* = +1 is ranked one standard deviation higher than the German reference, whereas, a *Δz* of -1 is ranked one standard deviation lower than the German reference. Mean values ± standard deviations are given.
(DOCX)

S3 Table Short Form 36 (SF36) transformed scores; mean values ± standard deviations are given: Comparison of healthy controls to patients with sacroiliac joint (SIJ) pain prior to pelvic belt application (pre). (DOCX)

S4 Table Short Form 36 (SF36) transformed scores; mean values ± standard deviations are given: Comparison of healthy controls to patients with sacroiliac joint (SIJ) pain in a six-week follow up of pelvic belt application (post). (DOCX)

S5 Table Muscle activation data: Inner-group comparison of the alterations within SIJs patient and controls related to moderate and maximum tension (*p-*values refer to the RMS data given in Table 4). (DOCX)

S6 Table Muscle activation data: Comparison of the relative variability of muscle activation in SIJ patients and controls in the conditions without pelvic belt, under moderate and maximum belt tension. (DOCX)

S7 Table Muscle activation data: Inner-group comparison of the variability in muscle activation within SIJ patients and controls without pelvic belt application, under moderate and maximum tension (*p-*values refer to the variability data given in S6 Table). (DOCX)
