# Correction: Pelvic Belt Effects on Health Outcomes and Functional Parameters of Patients with Sacroiliac Joint Pain

**DOI:** 10.1371/journal.pone.0140090

**Published:** 2015-10-02

**Authors:** Niels Hammer, Robert Möbius, Stefan Schleifenbaum, Karl-Heinz Hammer, Stefan Klima, Justin S. Lange, Odette Soisson, Dirk Winkler, Thomas L. Milani

Table 2 is a duplicate of Table 3. Please view the correct Table 2 here.

**Table 2:** Short Form 36 (SF36) transformed scores of patients with sacroiliac joint (SIJ) pain prior to pelvic belt application (pre) and in a six-weeks follow up with pelvic belt application (post). Mean values ± standard deviations are given.

SF36 scores	SIJ patients pre (n = 17)	SIJ patients post (n = 15)	*p*
Physical functioning	56.2 ± 17.5	73.2 ± 17.5	***0*.*002***
Role functioning physical	63.2 ± 26.4	68.8 ± 26.5	*0*.*110*
Bodily pain	39.0 ± 21.0	50.7 ± 21.5	***0*.*006***
General health	58.8 ± 16.3	55.4 ± 14.1	*0*.*283*
Vitality	50.0 ± 13.7	51.3 ± 12.8	*0*.*551*
Social functioning	69.9 ± 26.2	72.5 ± 26.4	*0*.*353*
Role functioning emotional	78.1 ± 31.9	77.8 ± 26.7	*0*.*663*
Mental health	66.3 ± 16.8	68.0 ± 15.3	*0*.*435*
Physical summary	37.1 ± 5.9	41.7 ± 8.4	***0*.*013***
Mental summary	50.5 ± 9.7	48.3 ± 9.6	*0*.*345*

## References

[1] Hammer N, Möbius R, Schleifenbaum S, Hammer K-H, Klima S, Lange JS, et al (2015) Pelvic Belt Effects on Health Outcomes and Functional Parameters of Patients with Sacroiliac Joint Pain. PLoS ONE 10(8): e0136375 doi:10.1371/journal.pone.0136375 26305790 10.1371/journal.pone.0136375 PMC4549265

