# Supplementary material for: Pelvic Belt Effects on Health Outcomes and Functional Parameters of Patients with Sacroiliac Joint Pain
Source: PLoS One. 2015 Aug 25;10(8):e0136375. doi: 10.1371/journal.pone.0136375 (PMC4549265; doi:10.1371/journal.pone.0136375)
Supplement: S1 File — (PDF) [file pone.0136375.s001.pdf]

Ethik-Antrag zur Studie

Anatomisch-biomechanische Untersuchung der Wirkung der Kompression des Kreuzdarmbeingelenks auf das Kreuzdarmbeingelenkssyndrom durch Beckenorthesen

Als Anlagen sind folgende Unterlagen beigefügt

1. Wissenschaftlicher Hintergrund
2. Zielsetzung
3. Methoden an Probanden und Patienten
4. Ein- und Ausschlusskriterien
5. Patienten- und Probandeninformation zur Testung des Beckengurts, Patienteneinverständniserklärung
6. Literaturverzeichnis

1. Wissenschaftlicher Hintergrund

Zu den biomechanischen Aufgaben des Beckenrings zählen, Bewegung, Statik und Kraftübertragung gleichermaßen zu koordinieren. Das knöcherne Becken ohne die umgebenden Bandstrukturen verfügt über wenig Eigenstabilität. Pathologien des lumbosakralen Übergangs, wie etwa der tiefe Rückenschmerz und das Iliosakralgelenksyndrom (ISG-Syndrom), haben enorme volkswirtschaftliche Bedeutung (Wu et al., 2004; Papageorgiou et al., 1995). Durch die Bänder des Kreuzdarmbeingelenks (SL: Lig. sacroiliacum anterius = ASL, Lig. sacroiliacum interosseum = ISL, Lig. sacroiliacum posterius = PSL) verlaufen die Hauptkraftlinien des aufrechten Menschen. Der Einfluss des hinteren Bandapparats und die damit verbundenen Innervationsverhältnisse auf den sog. tiefen Rückenschmerz bzw. das Iliosakralgelenksyndroms (ISG-Syndrom) konnten noch nicht abschließend geklärt werden. Während bei Patienten mit Beckenringfrakturen die operative Stabilisierung im Vordergrund therapeutischer Bemühungen steht (Tscherne et al., 1998), werden Patienten mit tiefem Rückenschmerz oder ISG-Syndrom oftmals minimalinvasiv durch Infiltration vermeintlich beteiligter Facettengelenke mit Lokalanästhetika therapiert (Staal, 2010). Beckenorthesen stellen eine weitere konservative Maßnahme der Versorgung solcher Patienten dar. Untersuchungen an Patienten mit Beckenorthesen konnten belegen, dass sowohl in klinischen Tests (Mens et al., 1999), als auch in Bezug auf die neuronale Aktivierung der unteren Extremität durch die Kompression des Beckenrings abweichende Muster zu verzeichnen waren. Diese Beobachtungen und die durch die Patienten beschriebene subjektiv empfundene Besserung der Beschwerden unter der Anlage von Beckenorthesen (Ostgaard et al., 1994; Mens et al., 2006) legen die Vermutung nahe, dass sich ein morphologisches Korrelat im Bereich des ISG durch geeignete Bildgebung nachweisen lässt. Die Bänderverläufe im Bereich des ISG und die damit verbundenen Innervationsverhältnisse sind noch wenig untersucht. Die Bandstrukturen des SL gruppieren sich um das Gelenk und limitieren dessen Bewegung (Fick, 1904; Vrahas et al., 1995). Die technischen Voraussetzungen zur Vermessung, Visualisierung und Beschreibung der Bänder des ISG sind zwischenzeitlich gegeben (Attias et al., 2001; Steinke, 2001; Thomas et al., 2007; Steinke et al., 2008). Eigene Untersuchungen zeigen, dass sich für die Bänder des ISG reproduzierbare Formen und statistische Abhängigkeiten belegen lassen (Hammer et al., 2009; Hammer et al.; 2010; Steinke et al., 2010). Darum ist allein aus der Form bereits von einer differenzierten Funktion der Bänder auszugehen (Pauwels, 1960). Die SL stehen im Fokus unfallchirurgischer Forschung (Baumgärtel, 1998; Pohlemann et al., 1996; Culemann et al., 2004). Inzwischen stehen neben Gefrierschnitttechniken (Fick, 1904), Plastinationstechniken (Steinke, 2001) und histologischen Untersuchungen für Körperspender auch CT und MRT für die klinische Anwendung zur Verfügung. Hiermit können Aussagen über die SL in-situ getroffen werden (Puhakka et al., 2004; Steinke et al., 2010). Radiologisch akquirierte Bilddaten stellen die Grundlage für die Rekonstruktion dreidimensionaler Patientenmodelle dar. Radiologen und Anatomen sind aufgrund ihrer anatomischen Kenntnisse und aufgrund ihres fachlichen Trainings in der Lage, die Bilddaten durch geeignete Werkzeuge zu segmentieren.

2. Zielsetzung

Ziel des Projekts ist die Untersuchung des Einflusses der Beckenkompression durch die Orthese SacroLoc (Bauerfeind AG, Zeulenroda-Triebes, Deutschland) auf die Kompression des Beckenrings und Schmerzreduktion beim tiefen Rückenschmerz und ISG-Syndrom. Die anatomischen Schwerpunkte liegen auf der Darstellung der Innervationsverhältnisse und der Lagebeziehung der Gelenkflächen und Bandstrukturen des ISG unter besonderer Berücksichtigung des Einflusses der Kompression durch die Beckenorthese. Das anatomische und funktionelle Korrelat wird in vivo an 20 Patienten mit bestehendem ISG-Syndrom und Besserung der Beschwerden unter der Anlage der Beckenorthese überprüft: MRT-Untersuchungen, Ganganalysen und EMG werden in drei Kompressionsstufen der Beckenorthese angefertigt. An 20 gesunden Probanden werden vergleichsweise MRT, Ganganalysen und EMG unter drei Kompressionsstufen der Orthese angefertigt. Die Befunde der anatomischen und biomechanischen Untersuchungen und die MRT-Bildgebung werden in eine computerbasierte Finite-Elemente-Simulation integriert, um eine virtuelle Validierung der Untersuchungsergebnisse vorzunehmen und Möglichkeiten der Optimierung einer Orthese aufzuzeigen.

3. Methoden an Probanden und Patienten

3.1 3 Tesla MRT

Veränderungen der Gelenkstrukturen, der Muskeln, Sehnen und Bänder spielen insbesondere in Orthopädie und Unfallchirurgie eine zentrale Rolle. Die MRT stellt aufgrund ihrer hohen Auflösung und des hohen Kontrasts in frei wählbarer Schichtorientierung ein besonders wertvolles diagnostisches Verfahren dar. Im Vergleich zu anderen bildgebenden Verfahren wie die Sonographie, Röntgen und CT bieten MRT die Vorteile nicht vorhandener Strahlenbelastung, des hervorragenden Weichteilkontrasts und der frei wählbaren Schichtorientierungen (Shellock and Kanal 1991), welche die Nachteile der räumlichen Enge und der Geräuschentwicklung zu kompensieren vermögen (Straub, Mack et al. 2006). Studien, die sich mit den Auswirkungen von MRT auf den Menschen befassen, beschränken sich hauptsächlich auf die entstehende Wärmeentwicklung (Shellock and Kanal 1991). In 1,5 T MRT konnte gezeigt werden, dass beobachtete Temperaturerhöhungen keine schädlichen Auswirkungen verursachen (Shellock und Schaefer, 1989; Shellock und Kanal, 1991). Von Klitzing konnte zwar Einflüsse statischer MRT Magnetfelder auf den Menschen belegen, ohne jedoch hieraus schädliche Einflüsse ableiten zu können. Hong und Shellock machten somatosensorischen Effekte zum Gegenstand ihrer Forschung und konnten auch hier keine Effekte auf den menschlichen Körper nachweisen (Hong and Shellock 1990). Im Zusammenhang mit Gradientenfeldern der MRT ist außerdem die Induktion elektrischer Ströme in leitendem Gewebe eine mögliche Gefahrenquelle, welche durch die 26. Verordnung des Bundesimmissionsschutzgesetzes verbindlich geregelt wird: Die Strahlenschutzkommission erließ Grenzwerte für die Exposition mit elektromagnetischer Strahlung, welche sich an internationalen Vorgaben orientieren (International Commission on Non-Ionizing Radiation Protection; ICNIRP 2010). Die Grenzwerte für beruflich Exponierte gelten auch für Patienten, die sich MRT unterziehen. Maßgeblich an der biologischen Wirkung hochfrequenter Strahlung beteiligt ist die vom Körper aufgenommene Strahlungsleistung. Diese bewirkt eine Temperaturerhöhung des Gewebes. International anerkannt als Größe für die

thermische Wirkung ist die sog. spezifische Absorptionsrate. Gegenwärtig werden vom Gesetzgeber für die allgemeine Bevölkerung Begrenzungen der spezifischen Absorptionsrate von 0,08 W/kg (über den ganzen Körper gemittelt) gefordert; für Teilkörperbereiche sind 2 W/kg zulässig. Für berufliche Exposition gelten 0,4 W/kg für den gesamten Körper, für Teilkörperbereiche 10 W/kg und für Extremitäten sogar 20 W/kg. Die SSK nimmt dabei die Grenzwerte der ICNIRP als Grundlage (Herbertz, 1998). Zusammenfassend kann festgestellt werden, dass eine gesundheitliche Schädigung bei Einhaltung der gesetzlichen Vorgaben nicht nachgewiesen werden kann. Hohe Feldstärken bedingen schnelle Untersuchungszeiten und eine gute Bildqualität vor allem durch das dadurch verbesserte Signal-zu-Rausch-Verhältnis (Soher et al., 2007; Straub et al., 2006).

3.2 Ganganalysen und Elektromyographie (geändert, 04.04.2011)

Analysen des menschlichen Gangbilds stellen ein leistungsfähiges Instrument dar, um den Beckenring und die untere Extremität in Abhängigkeit von Alter, Geschlecht, Gewicht und Pathologie zu untersuchen (Simon, 2004) und sind gleichzeitig gesundheitlich unbedenklich für die Teilnehmer. Elektromyographische Testungen (EMG) erlauben es zudem, Rückschlüsse auf muskuläre Aktivierungsmuster zu ziehen (Kleissen et al., 1998; Raez et al., 2006). Insbesondere die EMG-Ableitungen mittels Oberflächenelektroden stellen ein nicht invasives Verfahren dar. Diese werden im Gegensatz, zum Beispiel zu Feinnadelelektroden, nicht in das Muskelgewebe eingestochen, sondern auf die Haut geklebt. Es ist lediglich eine intensive Reinigung der Hautoberfläche notwendig, um die Elektroden zu befestigen. Daher besteht keinerlei gesundheitliche Gefahr für Patienten oder Probanden.

Durch die Kombination von EMG und Ganganalysen lassen sich aktive Rückschlüsse über die Funktionsmuster der unteren Extremität ableiten. Somit sind Einzelmuskeln als Ursache für verändertes Gangverhalten identifizierbar.

4. Ein- und Ausschlusskriterien

Der Einschluss der Probanden und Patienten erfolgt nach ausführlicher Aufklärung, der Überprüfung von Ein- und Ausschlusskriterien und durch ihr freiwilliges Einverständnis.

	Einschlusskriterien	Ausschlusskriterien
Patienten	- Eignung zur Durchführung der bildgebenden Diagnostik (MRT)<br>- ausreichende Konstitution und Koordination zur Durchführung der Ganganalysen (incl. Einbeinstand)	- Skelettabnormalitäten wie Rotationsfehlstellungen: z.B. pathologische Anteversion des Femur („in-toing“)<br>- Pathologien des Gangbilds (Erkrankungen des neuromuskulären Formenkreises)<br>- Einschränkungen der Gelenkbeweglichkeit (Kontrakturen), Arthrose, Arthritis, pathologische Gelenkstellungen und Gelenkformen, chronische Schmerzen außerhalb des Kreuzdarmbeingelenks oder des lumbosakralen Übergangs<br>- Verringerung der Muskelkraft (Kraftgrad < 5 nach Oxford / Janda)<br>- Infektion, Fieber<br>- Frakturen, Bandverletzungen, Muskelverletzungen, Weichteilschäden<br>- Herz-Kreislauferkrankungen<br>- Metallimplantate jeder Art, Herzschrittmacher, Defibrillatoren, Schmerzpumpen, Neurostimulatoren, Stents<br>- Platzangst, Geräuschangst<br>- somatoforme Erkrankungen, schwebende Berentungsverfahren<br>**- Schwangerschaft (geändert, 04.04.2011)**
Probanden	- siehe Einschlusskriterien Patienten	- siehe Ausschlusskriterien Patienten, zusätzlich:<br>- keine Erkrankungen des Bewegungsapparats, insbesondere des Kreuzdarmbeingelenks oder der Wirbelsäule

5. Patienten- und Probandeninformation zur Testung des Beckengurts

Sehr geehrte Patienten und Probanden,

Ziel der durchzuführenden Untersuchung ist die Überprüfung des Wirkmechanismus und der Wirkstärke eines Beckengurts (Beckenorthese). Hierzu werden Untersuchungen vor und nach dem Anlegen des Beckengurts durchgeführt. Zum Einsatz kommen Ganganalysen, Elektromyographie (EMG) und die Untersuchung mit Magnetresonanztomographie. Vor und nach der Untersuchung werden Sie gebeten, einen Fragebogen zur von Ihnen empfundenen Wirkung des Beckengürtels auf das Gangverhalten und die Schmerzreduktion (Patienten) auszufüllen. Bitte erscheinen Sie ausgeruht und ausreichend gesättigt in leichter Kleidung, möglichst in Sportsachen, und in geeignetem Schuhwerk (Turnschuhen).

Ablauf:

Während der Durchführung der Ganganalysen werden von wichtigen Muskeln des Beckens und der unteren Extremität Aufnahmen der elektrischen Aktivität (EMG) angefertigt. Hierfür ist es notwendig, die Messelektroden auf der Hautoberfläche durch geeignete Klebestreifen auf der Haut zu befestigen. Bei starker Behaarung kann es notwendig sein, vor dem Befestigen der Klebeelektroden des EMG die Hautareale (Größe einer 50 Cent Münze) frei zu rasieren. Weiterhin werden die Hautareale mit einer entfettenden, hautfreundlichen Lösung gereinigt, um eine störungsfreie Übertragung der Messdaten sicherzugestellen. Die Ganganalysen werden in von Ihnen gewohntem Schuhwerk (Turnschuhe) oder gegebenenfalls ohne Schuhe durchgeführt: Hierzu ist es notwendig, wiederholt über eine Bodenmatte zu laufen, welche den Abdruck des Fußes beim Gehen vermisst und aufzeichnet. Zusätzlich erfolgt die Messung des Fußabdrucks auf der Datenmatte im Einbeinstand beider Seiten. Die Anfertigung der Ganganalysen nimmt ca. 60 Minuten in Anspruch. Die Messungen auf der Bodenmatte müssen mehrfach wiederholt werden. Zusätzlich zu den Ganganalysen erfolgen Schichtaufnahmen durch Magnetresonanztomographie (MRT). Es werden drei MRT-Sequenzen des Beckens, der Lendenwirbelsäule und der unteren Extremität angefertigt: ohne die Anwendung des Beckengurts, in geringer sowie in starker Straffung des Gürtels. Die Aufnahmen der Schnittbilder nehmen etwa 25 bis 35 Minuten pro Sequenz in liegender Körperposition in Anspruch.

Die MRT, Ganganalysen und EMG sind schmerzfreie, nicht gesundheitsschädliche und nicht invasive Messverfahren.

Für Ihre Fragen stehen wir Ihnen jederzeit zur Verfügung.

Geändert, 04.04.2011

Patienteneinwilligungserklärung

- Anatomisch-biomechanische Untersuchung der Wirkung der Kompression des Kreuzdarmbeingelenks auf das Kreuzdarmbeingelenkssyndrom durch die Orthese SacroLoc -

………………………………………………………… ……………………………………………………………
Name des Patienten Geb.datum

Ich bin in einem persönlichen Gespräch ausführlich und verständlich über Wesen, Bedeutung, Risiken und Tragweite der klinischen Studie aufgeklärt worden. Ich habe darüber hinaus den Text der Patienteninformation gelesen und verstanden. Ich hatte Gelegenheit, mit dem Arzt über die Durchführung der klinischen Studie zu sprechen. Alle meine Fragen wurden zufrieden stellend beantwortet.

Ich hatte ausreichend Zeit, mich für die Teilnahme an oben genannter Studie zu entscheiden.

Mir ist bekannt, dass ich jederzeit und ohne Angabe von Gründen meine Einwilligung zur Teilnahme an der Studie zurückziehen kann.

Zudem wurde ich darüber informiert, dass bei Eintritt eines Schadens kein Versicherungsschutz besteht, insofern es sich nicht um ein fahrlässiges Verschulden des Studienleiters handelt.

Ich bin mit der im Rahmen der klinischen Studie erfolgten Aufzeichnung von anonymisierten Daten und der eventuellen Weitergabe dieser Daten an den Sponsor, die Fa. Bauerfeind, Zeulenroda, einverstanden.

Ich erkläre mich hiermit bereit, an der oben genannten klinischen Studie freiwillig teilzunehmen. Ich habe die Patienteninformation gelesen und jeweils ein Exemplar der Patienteninformation und –einwilligung erhalten.

………………………………………………….. ……………………………………………………………..
Ort, Datum Unterschrift des Patienten

……………………………………………………………..
Unterschrift des Arztes/ des Studienleiters

6. Literaturverzeichnis

Attias N, Arzani S, Duncan G, Taber KH, Hayman LA (2001): Sectional imaging anatomy: pelvic ring ligaments. J Comp Assist Tomogr 25: 975-979.

Baumgaertel F (1998): Tscherne Unfallchirurgie: Becken und Acetabulum / H. Tscherne (Hrsg.). Unter Mitarb. von F. Baumgaertel. Springer Verlag. Berlin, Heidelberg

Culemann U, Tosounidis G, Reilmann H, Pohlemann T (2004): Beckenringverletzung. Diagnostik und aktuelle Behandlungsmöglichkeiten. Der Unfallchirurg 107: 1169-1184.

Fick, R (1904): Anatomie der Gelenke: Kreuz-Darmbeingelenk. In: v. Bardeleben, K. (Ed.), Handbuch der Anatomie und Mechanik der Gelenke unter Berücksichtigung der bewegenden Muskeln. Gustav Fischer, Jena, pp. 289-303.

Hammer N, Steinke H, Böhme J, Stadler J, Josten C, Spanel-Borowski K (2010): Description of the iliolumbar ligament for computer-assisted reconstruction. Ann Anat 192: 162-167.

Hammer N, Steinke H, Slowik V, Josten C, Stadler J, Böhme J, Spanel-Borowski K (2009): The sacrotuberous and the sacrospinous ligament - A virtual reconstruction. Ann Anat. 191: 417-425.

Hong CZ, Shellock FG (1990): Short-term exposure to a 1.5 tesla static magnetic field does not affect somato-sensory-evoked potentials in man. Magn Reson Imaging 8: 65-69.

ICNIRP (2010): Guidelines for limiting exposure to time-varying electric and magnetic fields (1 Hz to 100 kHz). Health Phys 99(6):818-836.

Kleissen RF, Buurke JH, Harlaar J, Zilvold G (1998): Electromyography in the biomechanical analysis of human movement and its clinical application. Gait Posture. 8(2):143-158.

Mens JM, Damen L, Snijders CJ, Stam HJ (2006a): The mechanical effect of a pelvic belt in patients with pregnancy-related pelvic pain. Clinical Biomechanics 21(2):122–7.

Mens JM, Vleeming A, Snijders CJ, Stam HJ, Ginai AZ (1999): The active straight leg raising test and mobility of the pelvic joints. European Spine Journal 8(6):468–74.

Ostgaard HC, Zetherstrom G, Roos-Hansson E, Svanberg B (1994b): Reduction of back and posterior pelvic pain in pregnancy. Spine 19(8):894–900.

Papageorgiou AC, Croft PR, Ferry S, Jayson MI, Silman AJ (1995): Estimating the prevalence of low back pain in the general population. Evidence from the South Manchester Back Pain Survey. Spine 20: 1889-1894.

Pohlemann T, Tscherne H, Baumgartel F, Egbers HJ, Euler E, Maurer F, Fell M, Mayr E, Quirini WW, Schlickewei W, Weinberg A (1996): Pelvic fractures: epidemiology, therapy and long-term outcome. Overview of the multicenter study of the Pelvis Study Group. Unfallchirurg 99: 160-167.

Puhakka KB, Melsen F, Jurik AG, Boel LW, Vesterby A, Egund N (2004): MR imaging of the normal sacroiliac joint with correlation to histology. Skeletal Radiol 33: 15-28.

Raez MB, Hussain MS, Mohd-Yasin F (2006): Techniques of EMG signal analysis: detection, processing, classification and applications. Biol Proced Online. 8:11-35.

Shellock FG, Kanal E (1991): Policies, guidelines, and recommendations for MR imaging safety and patient management. SMRI Safety Committee. J Magn Reson Imaging 1: 97-101.

Shellock FG, Schaefer DJ, Crues JV (1989): Alterations in body and skin temperatures caused by magnetic resonance imaging: is the recommended exposure for radiofrequency radiation too conservative? Br J Radiol 742: 904-909.

Simon SR (2004): Quantification of human motion: gait analysis-benefits and limitations to its application to clinical problems. J Biomechanics, 1869–1880

Soher BJ, Dale BM, Merkle EM (2007): A review of MR physics: 3T versus 1.5T. Magn Reson Imaging Clin N Am 15: 277-290

Staal JB, de Bie R, de Vet HCW, Hildebrandt J, Nelemans P (2010): Injection therapy for subacute and chronic low-back pain (Review). The Cochrane Libary

Steinke H (2001): Plastinated body slices for verification of magnetic resonance tomography images. Ann. Anat. 183: 275-281.

Steinke H, Hammer N, Slowik V, Stadler J, Josten C, Böhme J, Spanel-Borowski K (2010): Novel insights into the sacro-iliac joint ligaments. Spine 35: 257-263.

Steinke H, Rabi S, Saito T (2008): Staining body slices before and after plastination. Europ J Anat. 12: 51-55

Straub R, Mack M, Jacobi V, Proschek D, Vogl T (2006): Die Magnetresonanztomografie in der Orthopädie. Der Orthopäde 35: 626-631.

Thomas M, Steinke H, Schulz T (2004): A direct comparison of MR images and thin-layer plastination of the shoulder in the apprehension-test position. Surg Radiol Anat. 26: 110-117.

Tscherne H, Regel G, Pape HC (1998): Internal fixation of multiple fractures in patients with polytrauma. Clin Orthop Relat Res 347: 62-78.

von Klitzing, L (1990): Effect of static magnetic fields on biosignal processing in humans. Biomed Tech (Berl) 35 Suppl 2: 17-19.

Vrahas M, Hern TC, Diangelo D, Kellam J, Tile M (1995): Ligamentous Contributions to Pelvic Stability. Orthopedics 18: 271-274.

Wu WH, Meijer OG, Uegaki K, Mens J, Van Diee¨n JH, Wuisman PIJM, O¨ stgaard HC (2004): Pregnancy-related pelvic girdle pain (pregnancy-related PGP), I: terminology, clinical presentation, and prevalence. Eur. Spine J. Epub. 13, 575–589.

Application for an ethics approval

Anatomical and biomechanical investigation of pelvic belt effect in sacroiliac joint dysfunction

Index:

1. Scientific Background
2. Aims and Objectives
3. Methods and Devices
4. Inclusion and exclusion criteria
5. Patient consent form
6. References (not translated)

1. Scientific Background (shortened)

- pelvic ring is involved in motion, force transmission

- sacroiliac joint dysfunction as a common disease with vast economic impact

- presently the role of the ligaments of the posterior pelvic ring in maintaining stability and nociceptors located inside have to date not been investigated thoroughly

- treatment of chronic sacroiliac joint dysfunction mostly based on injection of local anesthetics or surgical intervention

- pelvic belts (external pelvic compression belts) as one conservative approach to treat sacroiliac joint dysfunction, but lacking evidence on potential mechanisms of pain reduction

2. Aims and Objectives

- to compare bony alignment of pelvis and lumbar spine, sacroiliac joint alignment, muscle activation, gait patterns, pain intensity and health related quality of life between patients with sacroiliac joint dysfunction and healthy controls

- to determine the effects of pelvic belts in 20 patients with sacroiliac joint dysfunction and 20 healthy controls under different conditions of compression: moderate (as recommended by manufacturer) and maximum (just below pain perception related to the pelvic belt)

3. Methods and Devices

- bony alignment of pelvis and lumbar spine, sacroiliac joint: 3T magnetic resonance imaging

- muscle activity of gluteus maximus, tensor fasciae late, rectus femoris, vastus medialis, biceps femoris, adductors, tibialis anterior, gastrocnemius (medial head): surface EMG

- gait patterns: gait analysis plates with participants in one-leg stance and walking

- pain / pain related quality of life: NRS scale, short form 36 scale

4. Inclusion and exclusion criteria

Inclusion criteria:

SIJ patients: pain duration of at least twelve weeks, at least three positive SIJ pain provocation tests and if intra-articular injection of local anesthetics provided temporal relief of the symptoms

Controls: no history of musculoskeletal disorders

Exclusion criteria:

<table>
<tr><th>SIJ patients</th><th>Healthy controls</th></tr>
<tr><td colspan="2">fractures or muscular disorders</td></tr>
<tr><td colspan="2">metallic implants (e.g. pacemakers or endoprostheses)</td></tr>
<tr><td colspan="2">previous episodes of claustrophobia</td></tr>
<tr><td colspan="2">somatoform disorders</td></tr>
<tr><td colspan="2">pregnancy</td></tr>
<tr><td>degenerative joint diseases except sacroiliac joint pain</td><td>any kind of degenerative or inflammatory joint disease</td></tr>
<tr><td>inflammatory joint diseases</td><td>complaints of the low back and/or history of low back pain</td></tr>
</table>

5. Informed consent to participate in the research project

Anatomical and biomechanical investigation of pelvic belt effect in sacroiliac joint dysfunction

I hereby confirm that I was informed by the doctor Mr. / Ms. _______ orally about the nature, significance, implications and risks of the proposed study and had enough thinking time for my decision.

I have read the patient information and I feel sufficiently informed and I understand what it is about. The doctor has given me ample opportunity to ask questions, all of which were satisfactorily answered for me. I had enough time for my decision.

I understand that in scientific studies personal data and medical records are collected. The transfer, storage and analysis of this study-related data is done in accordance with legal provisions and sets that before participating in the study my voluntary consent is required:

1. I hereby agree that my personal data /patient history data will be recorded on questionnaires and electronic data carriers and passed without attribution to the competent surveillance authority (State Office or county government) or federal authority (Federal Institute for Drugs and Medical Devices, Bonn) for review the proper conduct of the study in as pseudonymous form.
2. I also declare that I agree that an authorized and committed secrecy officer of the competent domestic (and foreign) Surveillance Authority or the competent higher federal authority in my existing when the investigator personal data takes insight, so far as is necessary for the review of the study. For this measure, I release the investigator from the medical confidentiality.

My consent to participate in this research project as a patient or subject is done entirely voluntary.

I was advised that I can withdraw my consent without giving reasons at any time, without incurring any disadvantages for my further medical treatment and medical care.

I have a copy if this patient information and signed informed consent obtained.

Place, date Patient signature

Place, date Signature of the physician
