# Supplementary material for: Pelvic Belt Effects on Health Outcomes and Functional Parameters of Patients with Sacroiliac Joint Pain
Source: PLoS One. 2015 Aug 25;10(8):e0136375. doi: 10.1371/journal.pone.0136375 (PMC4549265; doi:10.1371/journal.pone.0136375)
Supplement: S1 Table — (DOCX) [file pone.0136375.s003.docx]

**S1 Table**

Extended baseline data of patients with sacroiliac joint (SIJ) pain, pain duration and numerical rating scale (NRS) data; BMI = body mass index, l = left, r = right, RMS = root mean square, mean values ± standard deviations are given

| **SIJ patient number** | | **Age** | | | **Gender** | **Height** | | | **Weight** | | | **BMI** | | | **Dominant** | **Pain** | | | **Numerical rating scale** | | | | | | | | | | | |
| --- | --- | --- | --- | --- | --- | --- | --- | --- | --- | --- | --- | --- | --- | --- | --- | --- | --- | --- | --- | --- | --- | --- | --- | --- | --- | --- | --- | --- | --- | --- |
|  |  |  | | |  |  |  |  |  |  |  |  | | | **leg** | **duration** | | | **last 2** | | | **no** | | | **moderate** | | | **maximum** | | |
|  |  | **[years]** | | |  | **[m]** | | | **[kg]** | | | **[kg/m^2^]** | | |  | **[months]** | | | **weeks** | | | **belt** | | | **tension** | | | | | |
|  | |  | | |  |  | | |  | | |  | |  |  |  | | |  | | |  | | |  | | |  | | |
| 1 | | 39 | | | f | 1.60 | | | 50.9 | | | 19.9 | | | r | 46 | | | 6 | | | 4 | | | 6 | | | 4 | | |
| 2 | | 45 | | | f | 1.64 | | | 69.3 | | | 25.8 | | | r | 82 | | | 10 | | | 8 | | | 8 | | | 8 | | |
| 3 | | 49 | | | m | 1.80 | | | 79.9 | | | 24.7 | | | r | 130 | | | 4 | | | 3 | | | 4 | | | 5 | | |
| 4 | | 71 | | | m | 1.75 | | | 86.5 | | | 28.2 | | | l | 170 | | | 7 | | | 2 | | | 1 | | | 5 | | |
| 5 | | 40 | | | f | 1.78 | | | 91.3 | | | 28.8 | | | r | 36 | | | 4 | | | 3 | | | 5 | | | 4 | | |
| 6 | | 33 | | | f | 1.53 | | | 58.0 | | | 24.8 | | | r | 12 | | | 4 | | | 4 | | | 3 | | | 4 | | |
| 7 | | 39 | | | f | 1.78 | | | 64.2 | | | 20.3 | | | l | 54 | | | 4 | | | 3 | | | 2 | | | 3 | | |
| 8 | | 46 | | | m | 1.87 | | | 85.3 | | | 24.4 | | | r | 54 | | | 6 | | | 4 | | | 4 | | | 3 | | |
| 9 | | 55 | | | m | 1.68 | | | 88.4 | | | 31.3 | | | l | 18 | | | 4 | | | 6 | | | 0 | | | 4 | | |
| 10 | | 51 | | | f | 1.67 | | | 61.1 | | | 21.9 | | | l | 4 | | | 7 | | | 6 | | | 5 | | | 6 | | |
| 11 | | 32 | | | m | 1.74 | | | 66.9 | | | 22.1 | | | r | 19 | | | 3 | | | 2 | | | 0 | | | 0 | | |
| 12 | | 46 | | | m | 1.73 | | | 73.4 | | | 24.5 | | | r | 10 | | | 6 | | | 6 | | | 4 | | | 3 | | |
| 13 | | 60 | | | f | 1.63 | | | 68.8 | | | 25.9 | | | r | 60 | | | 4 | | | 3 | | | 3 | | | 5 | | |
| 14 | | 42 | | | f | 1.75 | | | 73.3 | | | 23.9 | | | l | 120 | | | 3 | | | 2 | | | 2 | | | 3 | | |
| 15 | | 27 | | | m | 1.84 | | | 71.6 | | | 21.1 | | | r | 4 | | | 3 | | | 2 | | | 2 | | | 1 | | |
| 16 | | 38 | | | f | 1.64 | | | 83.1 | | | 30.9 | | | r | 12 | | | 7 | | | 6 | | | 5 | | | 6 | | |
| 17 | | 54 | | | f | 1.68 | | | 71.8 | | | 25.4 | | | r | 96 | | | 5 | | | 5 | | | 4 | | | 4 | | |
|  |  |  | | |  |  | | |  | | |  | | |  |  | | |  | | |  | | |  | | |  | | |
|  | SIJ patients | 45.1 | ± | 11.0 |  | 1.71 | ± | 0.09 | 73.2 | ± | 11.3 | 24.9 | ± | 3.4 |  | 54.5 | ± | 49.7 | 5.0 | ± | 1.9 | 4.0 | ± | 1.8 | 3.4 | ± | 2.1 | 4.0 | ± | 1.9 |
|  | controls | 43.7 | ± | 19.9 |  | 1.68 | ± | 0.10 | 68.1 | ± | 9.3 | 24.2 | ± | 3.9 |  |  |  |  |  |  |  |  |  |  |  |  |  |  |  |  |
