# Supplementary material for: Pelvic Belt Effects on Health Outcomes and Functional Parameters of Patients with Sacroiliac Joint Pain
Source: PLoS One. 2015 Aug 25;10(8):e0136375. doi: 10.1371/journal.pone.0136375 (PMC4549265; doi:10.1371/journal.pone.0136375)
Supplement: S2 Table — A Δz = +1 is ranked one standard deviation higher than the German reference, whereas, a Δz of -1 is ranked one standard deviation lower than the German reference. Mean values ± standard deviations are given. (DOCX) [file pone.0136375.s004.docx]

**S2 Table**

*Z*-transformed values of the short form 36 (SF-36) survey obtained from healthy controls and patients with sacroiliac joint (SIJ) pain prior to pelvic belt application (pre) and in a six-weeks follow up, referenced by the German population. A *Δz* = +1 is ranked one standard deviation higher than the German reference, whereas, a *Δz* of -1 is ranked one standard deviation lower than the German reference. Mean values ± standard deviations are given.

| **SF36 *z*-values** | **Controls**  *(n=17)* | | | **SIJ patients pre**  *(n=17)* | | | **SIJ patients follow up**  *(n=15)* | | |
| --- | --- | --- | --- | --- | --- | --- | --- | --- | --- |
|  |  | | |  | | |  | | |
| Physical functioning | 0.51 | ± | 0.25 | -1.24 | ± | 0.76 | -0.49 | ± | 0.76 |
| Role functioning physical | 0.15 | ± | 0.37 | -0.53 | ± | 0.78 | -0.37 | ± | 0.78 |
| Bodily pain | 0.60 | ± | 0.63 | -1.55 | ± | 0.89 | -1.05 | ± | 0.91 |
| General health | -0.03 | ± | 0.81 | -0.67 | ± | 0.81 | -0.84 | ± | 0.70 |
| Vitality | 0.12 | ± | 0.73 | -0.53 | ± | 0.66 | -0.47 | ± | 0.61 |
| Social functioning | 0.45 | ± | 0.75 | -0.61 | ± | 1.17 | -0.50 | ± | 1.18 |
| Role functioning emotional | 0.27 | ± | 0.48 | -0.10 | ± | 0.97 | -0.11 | ± | 0.81 |
| Mental health | 0.19 | ± | 0.68 | -0.48 | ± | 0.93 | -0.38 | ± | 0.85 |
