# Supplementary material for: Pelvic Belt Effects on Health Outcomes and Functional Parameters of Patients with Sacroiliac Joint Pain
Source: PLoS One. 2015 Aug 25;10(8):e0136375. doi: 10.1371/journal.pone.0136375 (PMC4549265; doi:10.1371/journal.pone.0136375)
Supplement: S3 Table — (DOCX) [file pone.0136375.s005.docx]

**S3 Table**

Short Form 36 (SF36) transformed scores; mean values ± standard deviations are given: Comparison of healthy controls to patients with sacroiliac joint (SIJ) pain prior to pelvic belt application (pre)

| **SF36 scores** | **controls** | | | **SIJ patients pre** | | | ***p*** |
| --- | --- | --- | --- | --- | --- | --- | --- |
|  | **(n=17)** | | | **(n=17)** | | |  |
| Physical functioning | 96.3 | ± | 5.8 | 56.2 | ± | 17.5 | ***0.000*** |
| Role functioning physical | 86.1 | ± | 12.4 | 63.2 | ± | 26.4 | ***0.040*** |
| Bodily pain | 89.6 | ± | 14.9 | 39.0 | ± | 21.0 | ***0.000*** |
| General health | 71.7 | ± | 16.4 | 58.8 | ± | 16.3 | *0.280* |
| Vitality | 63.5 | ± | 15.2 | 50.0 | ± | 13.7 | ***0.011*** |
| Social functioning | 93.8 | ± | 16.7 | 69.9 | ± | 26.2 | ***0.004*** |
| Role functioning emotional | 90.3 | ± | 15.7 | 78.1 | ± | 31.9 | *0.181* |
| Mental health | 78.2 | ± | 12.2 | 66.3 | ± | 16.8 | *0.023* |
|  |  |  |  |  |  |  |  |
| Physical summary | 53.6 | ± | 4.2 | 37.1 | ± | 5.9 | ***0.000*** |
| Mental summary | 51.7 | ± | 7.3 | 50.5 | ± | 9.7 | *0.706* |
