# Supplementary material for: Pelvic Belt Effects on Health Outcomes and Functional Parameters of Patients with Sacroiliac Joint Pain
Source: PLoS One. 2015 Aug 25;10(8):e0136375. doi: 10.1371/journal.pone.0136375 (PMC4549265; doi:10.1371/journal.pone.0136375)
Supplement: S4 Table — (DOCX) [file pone.0136375.s006.docx]

**S4 Table**

Short Form 36 (SF36) transformed scores; mean values ± standard deviations are given: Comparison of healthy controls to patients with sacroiliac joint (SIJ) pain in a six-weeks follow up with pelvic belt application

| **SF36 scores** | **controls**  *(n=17)* | | | **SIJ patients follow up**  *(n=15)* | | | ***p*** |
| --- | --- | --- | --- | --- | --- | --- | --- |
|  |  |  |  |  |  |  |  |
| Physical functioning | 96.3 | ± | 5.8 | 73.2 | ± | 17.5 | ***0.000*** |
| Role functioning physical | 86.1 | ± | 12.4 | 68.8 | ± | 26.5 | *0.031* |
| Bodily pain | 89.6 | ± | 14.9 | 50.7 | ± | 21.5 | ***0.000*** |
| General health | 71.7 | ± | 16.4 | 55.4 | ± | 14.1 | *0.006* |
| Vitality | 63.5 | ± | 15.2 | 51.3 | ± | 12.8 | *0.019* |
| Social functioning | 93.8 | ± | 16.7 | 72.5 | ± | 26.4 | *0.013* |
| Role functioning emotional | 90.3 | ± | 15.7 | 77.8 | ± | 26.7 | *0.124* |
| Mental health | 78.2 | ± | 12.2 | 68.0 | ± | 15.3 | *0.042* |
|  |  |  |  |  |  |  |  |
| Physical summary | 53.6 | ± | 4.2 | 41.7 | ± | 8.4 | ***0.000*** |
| Mental summary | 51.7 | ± | 7.3 | 48.3 | ± | 9.6 | *0.298* |
