# Supplementary material for: Pelvic Belt Effects on Health Outcomes and Functional Parameters of Patients with Sacroiliac Joint Pain
Source: PLoS One. 2015 Aug 25;10(8):e0136375. doi: 10.1371/journal.pone.0136375 (PMC4549265; doi:10.1371/journal.pone.0136375)
Supplement: S5 Table — (DOCX) [file pone.0136375.s007.docx]

**S5 Table**

Muscle activation data: Inner-group comparison of the alterations within SIJs patient and controls related to moderate and maximum tension (*p*-values refer to the RMS data given in Tab. 4)

| ***p-value*** | **ΔRMS_moderate tension_ : ΔRMS_maximum tension_** | | | |
| --- | --- | --- | --- | --- |
|  | **SIJ patients** | | **controls** | |
|  |  | |  | |
| **Muscle** |  |  |  |  |
| Biceps femoris | *0.878* | | *0.407* | |
| Gluteus maximus | *0.169* | | *0.287* | |
| Rectus femoris | ***0.007*** | | *0.877* | |
| Medial vastus | *0.959* | | *0.981* | |
