# Supplementary material for: Pelvic Belt Effects on Health Outcomes and Functional Parameters of Patients with Sacroiliac Joint Pain
Source: PLoS One. 2015 Aug 25;10(8):e0136375. doi: 10.1371/journal.pone.0136375 (PMC4549265; doi:10.1371/journal.pone.0136375)
Supplement: S6 Table — (DOCX) [file pone.0136375.s008.docx]

**S6 Table**

Muscle activation data: Comparison of the relative variability of muscle activation in SIJ patients and controls in the conditions without pelvic belt, under moderate and maximum belt tension. Mean values ± standard deviations are given.

|  | **variability_no belt_** | | | | | | | **variability_moderate tension_** | | | | | | | **variability_maximum tension_** | | | | | | |
| --- | --- | --- | --- | --- | --- | --- | --- | --- | --- | --- | --- | --- | --- | --- | --- | --- | --- | --- | --- | --- | --- |
|  | **SIJ patients** | | | **controls** | | | ***p*** | **SIJ patients** | | | **controls** | | | ***p*** | **SIJ patients** | | | **controls** | | | ***p*** |
|  |  |  |  |  |  |  |  |  |  |  |  |  |  |  |  |  |  |  |  |  |  |
| **Muscle** |  |  |  |  |  |  |  |  |  |  |  |  |  |  |  |  |  |  |  |  |  |
| Biceps femoris | 61.1% | ± | 52.1% | 52.2% | ± | 52.0% | *0.359* | 63.5% | ± | 41.7% | 48.9% | ± | 38.0% | *0.473* | 65.7% | ± | 50.3% | 49.1% | ± | 37.2% | *0.604* |
| Gluteus maximus | 32.3% | ± | 39.6% | 29.1% | ± | 22.9% | *0.359* | 19.5% | ± | 16.5% | 25.4% | ± | 11.5% | *0.141* | 46.3% | ± | 51.0% | 27.5% | ± | 20.3% | *0.824* |
| Rectus femoris | 46.1% | ± | 30.6% | 40.6% | ± | 48.2% | *0.660* | 46.0% | ± | 27.5% | 45.7% | ± | 43.8% | *0.897* | 46.0% | ± | 32.4% | 41.2% | ± | 37.5% | *0.979* |
| Medial vastus | 80.4% | ± | 61.3% | 64.5% | ± | 50.9% | *0.127* | 82.0% | ± | 56.4% | 44.7% | ± | 26.3% | *0.264* | 71.0% | ± | 46.1% | 58.8% | ± | 59.8% | *0.717* |
