# Supplementary material for: Pelvic Belt Effects on Health Outcomes and Functional Parameters of Patients with Sacroiliac Joint Pain
Source: PLoS One. 2015 Aug 25;10(8):e0136375. doi: 10.1371/journal.pone.0136375 (PMC4549265; doi:10.1371/journal.pone.0136375)
Supplement: S7 Table — (DOCX) [file pone.0136375.s009.docx]

**S7 Table**

Muscle activation data: Inner-group comparison of the variability in muscle activation within SIJ patients and controls without pelvic belt application, under moderate and maximum tension (*p*-values refer to the variability data given in *Table S4B*)

| ***p-value*** | **variability_no belt_ : variability_moderate tension_ : variability_maximum tension_** | | | |
| --- | --- | --- | --- | --- |
|  | **SIJ patients** | | **controls** | |
|  |  | |  | |
| **Muscle** |  |  |  |  |
| Biceps femoris | *0.670* | | *0.790* | |
| Gluteus maximus | *0.202* | | *0.838* | |
| Rectus femoris | *0.670* | | *0.943* | |
| Medial vastus | *0.741* | | *0.465* | |
